# Relationship between gut microbiota and multiple sclerosis: a scientometric visual analysis from 2010 to 2023

**DOI:** 10.3389/fimmu.2024.1451742

**Published:** 2024-08-19

**Authors:** Qingrong Ouyang, Hao Yu, Lei Xu, Ming Yu, Yunwei Zhang

**Affiliations:** ^1^ Department of Neurology, Suining Central Hospital, Suining, China; ^2^ Department of Emergency, Suining Central Hospital, Suining, China

**Keywords:** bibliometrics, gut microbiota, multiple sclerosis, scientometric, VOSviewer, CiteSpace

## Abstract

**Background:**

Numerous studies have investigated the relationship between gut microbiota (GM) and multiple sclerosis(MS), highlighting the significant role of GM in MS. However, there is a lack of systematic Scientometric analyses published in this specific research area to provide an overall understanding of the current research status.

**Methods:**

Perform a scientometric analysis on research conducted between 2010 and 2023 concerning the link between GM and MS using quantitative and visual analysis software (CiteSpace and VOSviewer.)

**Results:**

From January 1, 2010, and December 31, 2023, a total of 1019 records about GM and MS were retrieved. The number of publications exhibited a consistent upward trend annually. The United States led in publications, showed the strongest level of collaboration among countries. The University of California, San Francisco stands as the top institution in terms of output, and the most prolific and cited authors were Lloyd H. Kasper and Javier Ochoa-Reparaz from the USA. The research in this field primarily centers on investigating the alterations and associations of GM in MS or EAE, the molecular immunological mechanisms, and the potential of GM-based interventions to provide beneficial effects in MS or EAE. The Keywords co-occurrence network reveals five primary research directions in this field. The most frequently occurring keywords are inflammation, probiotics, diet, dysbiosis, and tryptophan. In recent years, neurodegeneration and neuropsychiatric disorders have been prominent, indicating that the investigation of the mechanisms and practical applications of GM in MS has emerged as a current research focus. Moreover, GM research is progressively extending into the realm of neurodegenerative and psychiatric diseases, potentially becoming future research hotspots.

**Conclusions:**

This study revealed a data-driven systematic comprehension of research in the field of GM in MS over the past 13 years, highlighted noteworthy research within the field, provided us with a clear understanding of the current research status and future trends, providing a valuable reference for researchers venturing into this domain.

## 1 Introduction

Multiple Sclerosis (MS) is a chronic inflammatory demyelinating disease of the central nervous system that is commonly caused by immune mediation, characterized by multiple lesions throughout the brain and spinal cord and dissemination in time, which results in a constellation of neurological impairments that eventually lead to accumulated disability. To date, MS remains one of the most common causes of neurological disability in young adults aged 18-40 (1). Worldwide, more than 2.5 million individuals are impacted by MS, imposing substantial burdens on both patients and their families. As we all know, the immunological dysregulation and self-reactive response that precipitate antibody attacks on CNS as the core pathology changes of MS. But the etiology of MS is highly heterogeneous and multifactorial, influenced by genetic and environmental factors (2), remains inadequately explained and with limited therapeutic options available, despite significant progress in the field of MS over the past two decades (3), including advancements in understanding basic immunological drivers, risk genes, and more effective treatment approaches.

Over the past decade, research on the GM in MS has been deepening. Increasing evidence showed that GM imbalance is linked to the pathogenesis of MS, and GM may serve as a potential preventive or therapeutic method for MS. The human gastrointestinal tract contains more than 100 trillion microorganisms, including bacteria, fungi, viruses, etc (4). These microorganisms have a significant impact on human health, such as aiding in food digestion and maintaining immune system balance (5). The human GM initially colonized from the mother and the environment, and shaped by diet and environmental factors such as lifestyle habits and sanitation conditions (6), which can reflect a certain genetic and environmental background. It is currently known that the human gut microbiome encodes over 3 million genes, producing thousands of metabolites (7). These substances facilitate bidirectional communication between the gastrointestinal system and the central nervous system (CNS) through the gut-brain axis, involving the vagus nerve and enteric nervous system, immune system, and microbial metabolites. They play a role in regulating endocrine, metabolic, immune, and neurotransmitter functions (8). Dysbiosis of the GM is linked to various neurological disorders such as developmental disabilities, neurodegeneration, and mood disorders (9). Numerous studies have noted alterations in the composition of the GM in patients with MS (10–14), and evidence from animal models indicates that alterations in GM, which trigger pro-inflammatory activation of the immune system, may represent a distinct disease mechanism for MS (15–17). Meanwhile, increasing number of studies focused on the pharmacology, dietary strategies, and other interventions related to GM and their positive impact on MS (18).

Bibliometric analysis is widely used to assess the development trend, research hotspots, academic influence, and discipline knowledge structure in a specific area through mathematical and statistical methods, and provides an important reference and decision-making basis for academic research (19). Over the past decade, there have been over a thousand studies published on the topic of GM in MS. However, bibliometric analysis focused on the field of GM in MS is still lacking. The present study utilizes VOSviewer and CiteSpace software to conduct scientometric analysis, summarizing the distribution and connections among authors, countries, and institutions, as well as keywords and references. The results of this analysis can assist researchers in identifying the current study status and trends, potential related journals, collaborators, and institutions, thereby enhancing the effectiveness of further work.

## 2 Materials and methods

### 2.1 Data source, cleaning, and extraction

**Figure 1:**
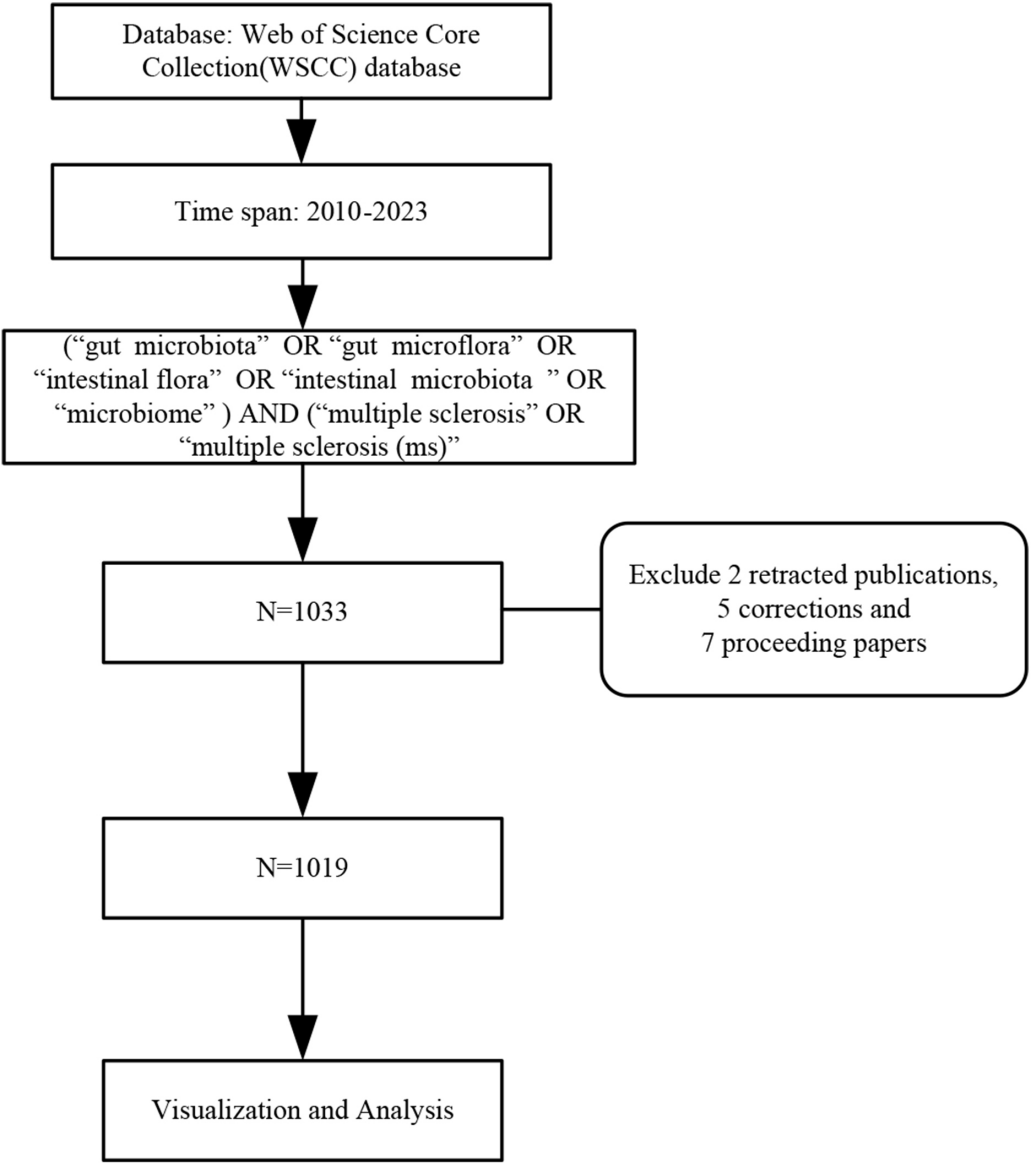
Flowchart of search strategy and data extraction.

The data source was obtained from the Web of Science Core Collection (WoSCC), publication period is set from January 1, 2010, to December 31, 2023. The search strategy was based on the mesh term combination listed below: “gut microbiota” OR “gut microflora” OR “intestinal flora” OR “intestinal microbiota” OR “microbiome” AND “multiple sclerosis” OR “multiple sclerosis (MS)”. We excluded 2 retracted publications, 5 corrections, and 7 proceeding papers from all the search documents, and the remaining 1019 results were retrieved, containing 421 articles, 485 reviews, 83 abstracts, 25 editorial material, 5 Letters. The search strategy is outlined in **Figure 1**.

### 2.2 Bibliometric and statistics analysis

The source data obtained from WoSCC were assessed and visually analyzed using various software tools including VOSviewer 1.6.19, Citespace 6.2.R6, Pajek, Scimago Graphica, RStudio, and Gephi, among others. Specifically, VOSviewer was employed to construct cooperative networks involving productive countries, institutions, authors, journals, and keywords. Citespace was utilized to identify prominent bursts in country/region publications and cited journals and to create temporal overlay cooperative network maps. The bibliometric package in RStudio was leveraged for analyzing trend topics, while other software tools were utilized for data processing. In this study, the threshold settings for selecting the top-ranking countries, institutions, authors, journals, and keywords are flexibly chosen based on the actual data extraction to ensure a comprehensive and accurate revelation of the relevant information in the GM in MS research field.

## 3 Results

### 3.1 Analysis of annual publication volume and trends

**Figure 2:**
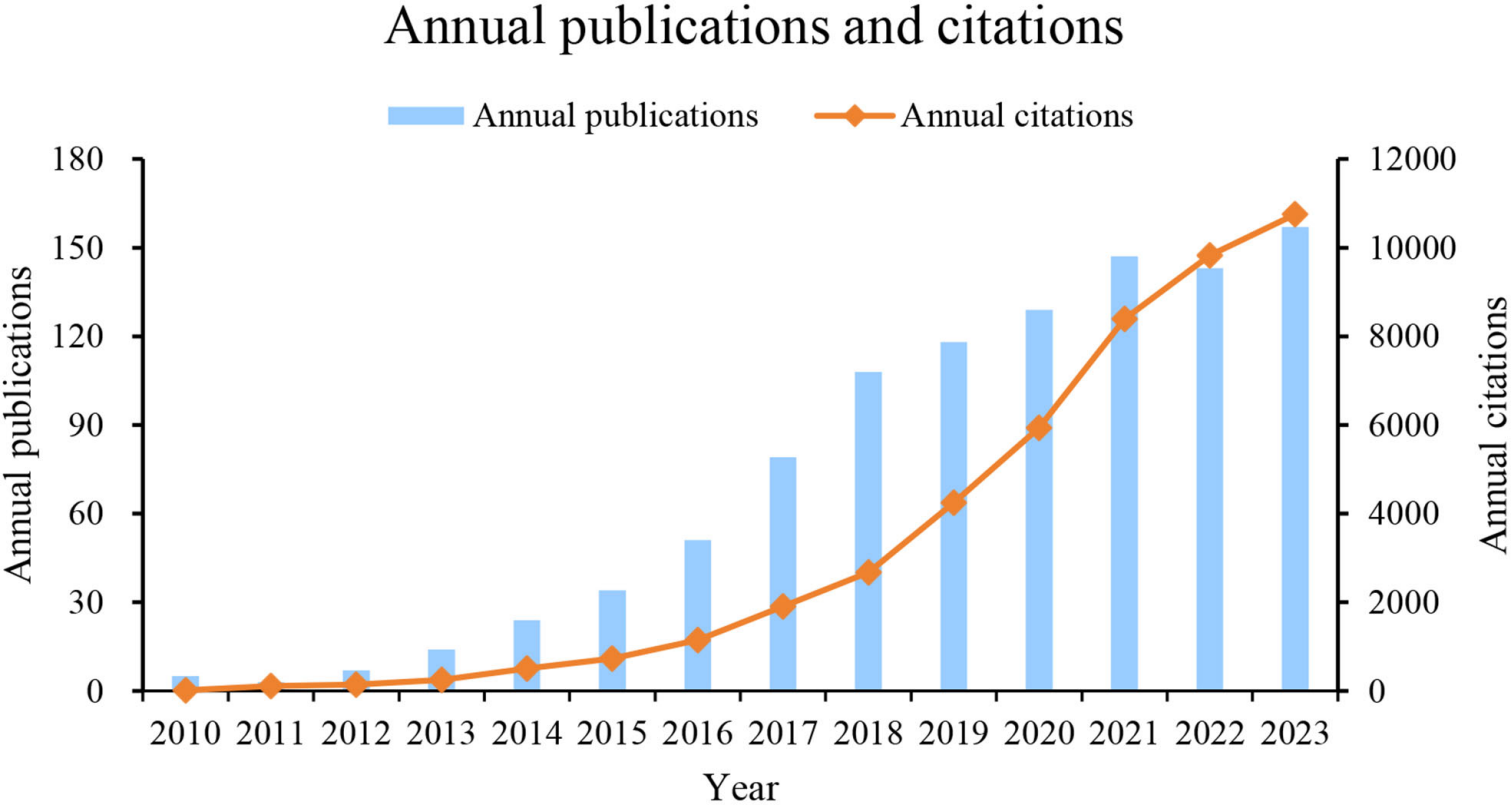
Annual publications and citations in GM and MS.

A total of 1019 papers meeting the search criteria were published from January 1, 2010, to December 31, 2023. The number of annual publications and citations on the relationship between GM and MS has shown an overall increasing trend over the years, as shown in **Figure 2**. From 2010 to 2013, there were steady growth rates with just single-digit publication numbers, with significant jumps in publication numbers in 2018, the annual publication volume exceeded one hundred for the first time. Although there were fluctuations in growth rates in some years, the general trend shows a positive trajectory. The data suggests a growing interest and research focus on the correlation between GM and MS, with a peak in publications in recent years (2021-2023).

### 3.2 Visual analysis of countries/regions distribution

**Figure 3:**
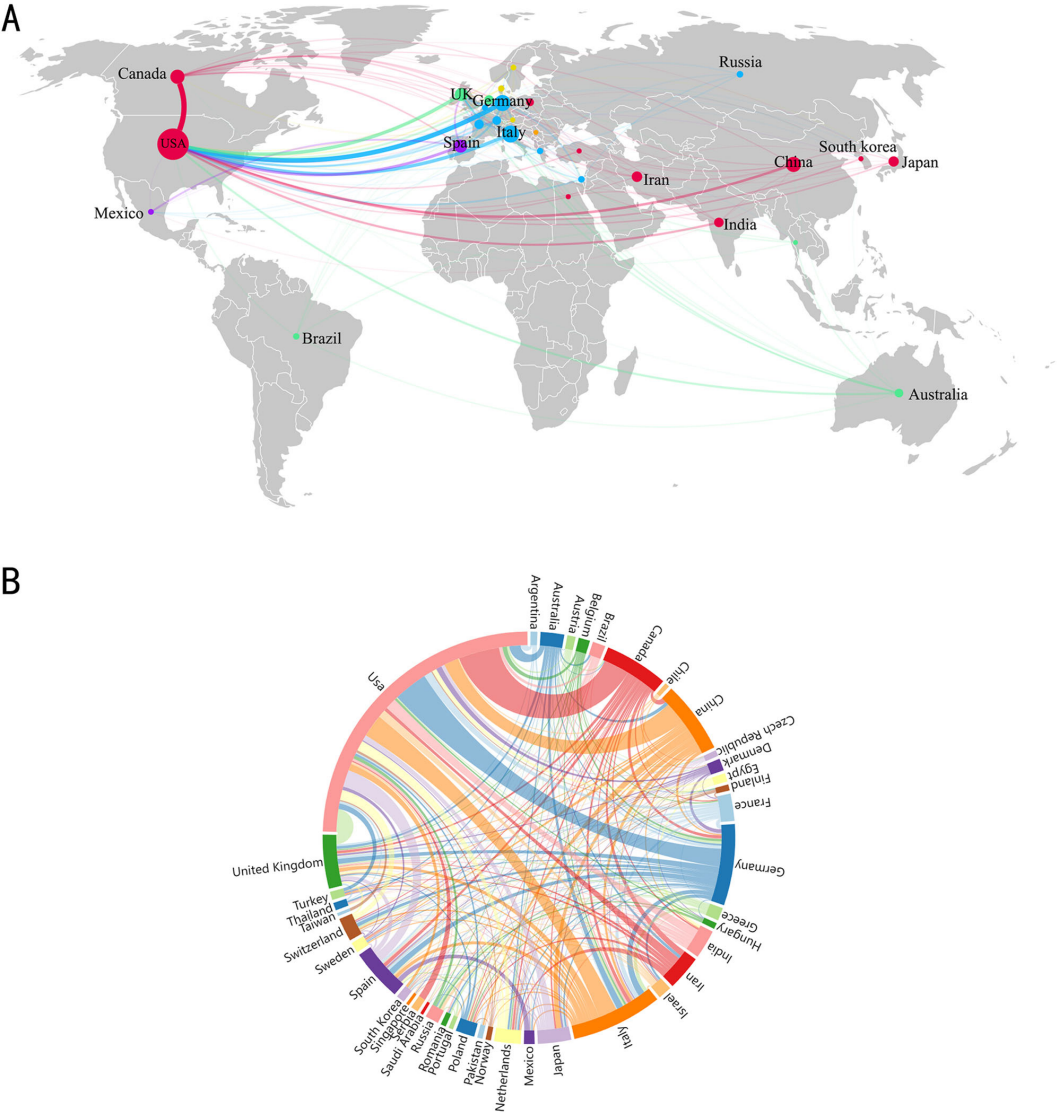
**(A)** Visualization of countries/regions analysis. **(B)** Country/region partnership chord maps.

Seventy countries/regions have contributed to the publication of research on the relationship between GM and MS. We established a minimum of 10 published documents per national, therefore 29 countries/regions satisfied the screening requirements. **Figures 3A, B** show the national distribution and collaboration diagram. In **Figure 3A**, each sphere represents a country, with the size of the circle indicating the number of publications, and the connecting lines between spheres illustrate clustering relationships in the study field, which are grouped into six clusters as depicted. In **Figure 3B**, the collaboration intensity between countries is further illustrated. The chord diagram represents each country with a curve segment, where longer segments indicate a higher number of publications from that country, the level of cooperation among nations is depicted by the thickness of the connecting lines; thicker lines signify a stronger collaboration. The most substantial collaborative strength in the field of GM and MS is observed between Canada and the USA with a weight of 36, This is closely followed by the robust collaboration between Germany and the USA, other notable collaborations include Italy and the USA, and the United Kingdom and the USA with weights of 20 each. These findings suggest a consistent trend of robust international cooperation within this research domain. The top 5 countries/regions by the number of publications are the USA with 393 publications (37.90%), Italy with 114 publications (10.99%), Germany with 99 publications (9.55%), China with 89 publications (8.58%), and Canada with 84 publications (8.10%). Collectively, these top 5 countries (USA, Italy, Germany, China, and Canada) contribute to around 72% of the total publications. However, there exists a significant disparity in publication numbers across different countries. While countries like the USA, Italy, and Germany lead the research in this area, others such as Iran, India, and Japan also make noteworthy contributions, albeit to a lesser extent. Moreover, regions like Brazil, Denmark, and Belgium show a smaller but growing presence in research pertaining to GM and MS. These regions are considered emerging and hold potential for further exploration and collaboration in this research domain.

### 3.3 Visual analysis of research institutions

**Figure 4:**
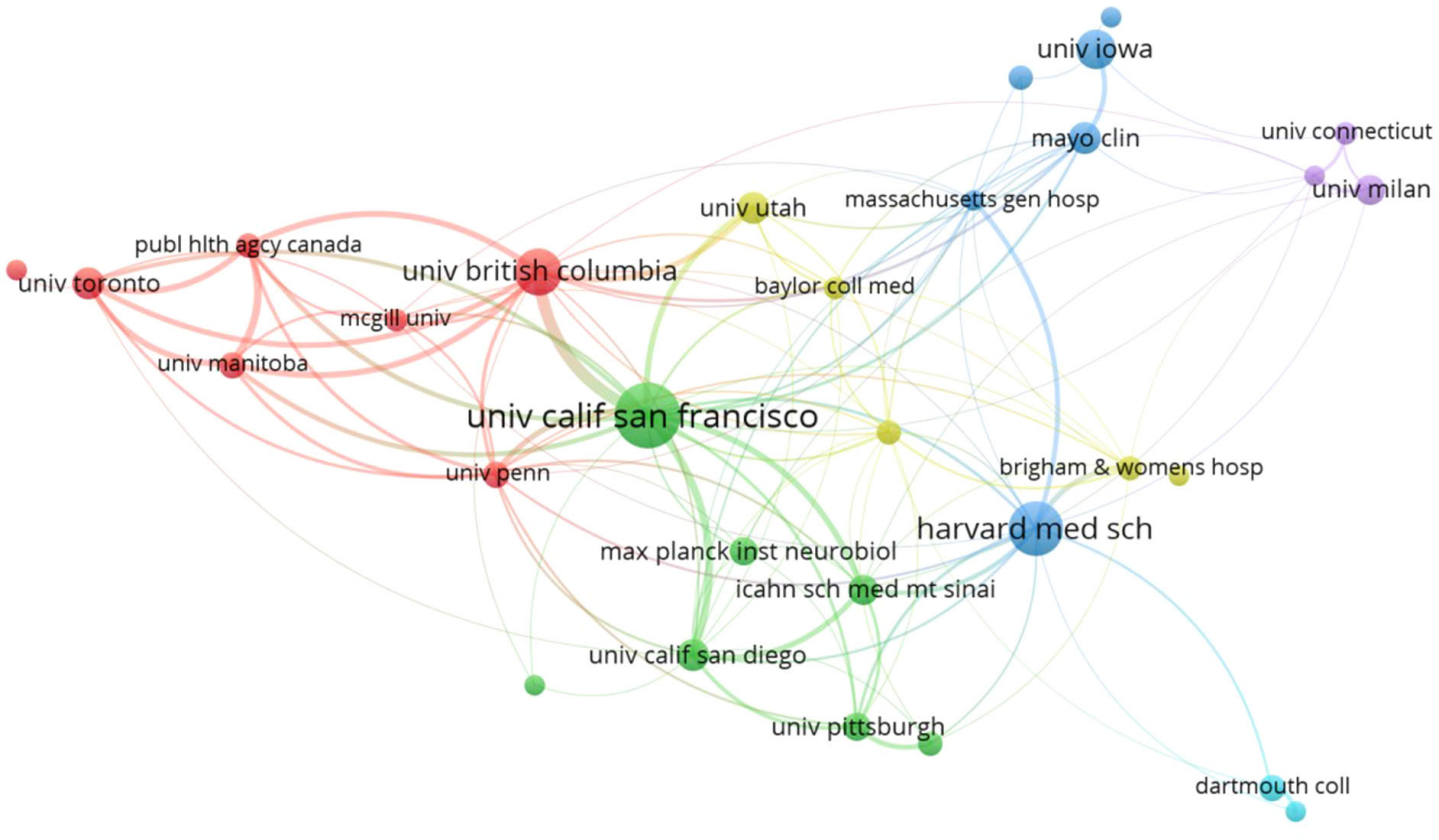
The collaboration network of research institutions.

Studies investigating the association between GM and MS have been conducted by 1581 distinct institutions. By applying a minimum publication threshold of 10 documents per institution, we generated a cooperation relationship and clustering map for the top 33 research institutes. The map illustrates collaboration patterns among institutions and categorizes them into six clusters, shown in **Figure 4**. The University of California, San Francisco, and Harvard Medical School topped the list with the highest document counts, published 57 and 43 documents, respectively. Following closely were the University of British Columbia, University of Iowa, Mayo Clinic, University of California, San Diego, University of Utah, University of Toronto, Icahn School of Medicine at Mount Sinai, and University of Milan, universities make up the majority of the institutions that produce the most research on GM and MS. Based on the cooperation network analysis, the University of California, San Francisco demonstrates prominent centrality in collaborating with various institutions.

### 3.4 Visual analysis of authors

**Figure 5:**
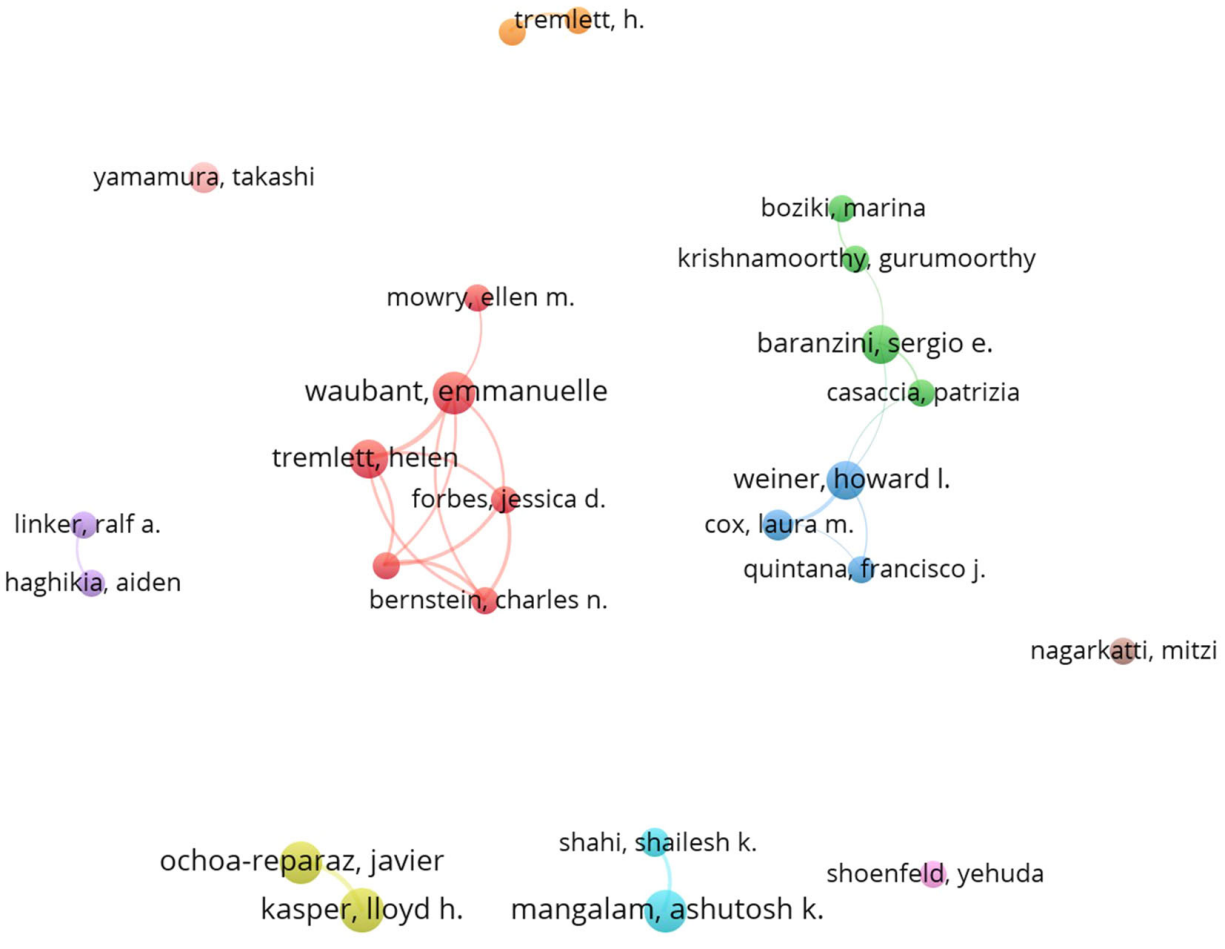
Visualization analysis of the cooperation network of research authors.

A total of 5185 authors contributed to publications related to the GM and MS. We set the criterion that each author published a minimum of 8 documents, a total of 24 authors were selected and incorporated into the co-author network, as shown in **Figure 5**. The size of each sphere in the image corresponds directly to the number of papers published by each author, the different clusters are identified by different colors. The network was divided into eleven clusters. The level of author collaboration is illustrated by the thickness of the lines linked to the spheres. Kasper, Lloyd H., and Ochoa-Reparaz, Javier have the highest number of publications, but their willingness to collaborate with other authors is notably weaker compared to the publications closely followed by Waubant, Emmanuelle, and Tremlett, Helen, indicated that the latter are actively engaged in collaborative efforts in the GM and MS field.

**Table 1** lists the top 15 authors ranked by the number of publications. The data indicates that most of the top authors are from the USA, with some representation from Canada, Japan, and Germany. This suggests a dominant presence of authors from prestigious institutions in the USA. Kasper, Lloyd H. leads in both the number of publications and total citations with 2245, followed by Krishnamoorthy, Gurumoorthy from Max Planck Inst Biochem with 1663 citations and Weiner, Howard L. from Harvard Med Sch with 1788 citations.

**Table 1:** Top authors with the highest number of articles and total citations.

NO.	Author	Country/region	Affiliation	Research articles	Reviews	Total citations of publications
1	Kasper, Lloyd H.	USA	Dartmouth Coll	12	8	2245
2	Ochoa-Reparaz, Javier	USA	Dartmouth Med Sch	13	6	1607
3	Waubant, Emmanuelle	USA	Univ Calif San Francisco	13	5	1085
4	Mangalam, Ashutosh K.	USA	Univ Iowa	8	6	923
5	Baranzini, Sergio E.	USA	Univ Calif San Francisco	14	3	1371
6	Tremlett, Helen	Canada	Univ British Columbia	9	4	632
7	Weiner, Howard L.	USA	Harvard Med Sch	10	3	1788
8	Cox, Laura M.	USA	Harvard Med Sch	8	2	1264
9	Yamamura, Takashi	Japan	Natl Inst Neurosci	4	2	731
10	Shahi, Shailesh K.	USA	Univ Iowa	7	1	487
11	Bernstein, Charles N.	Canada	Univ Manitoba	5	4	676
12	Forbes, Jessica D.	Canada	Univ Manitoba	5	4	676
13	Haghikia, Aiden	Germany	Ruhr Univ Bochum	5	4	817
14	Krishnamoorthy, Gurumoorthy	Germany	Max Planck Inst Biochem	5	4	1663
15	Tremlett, H.	Canada	Univ British Columbia	1	0	213

**Figure 6:**
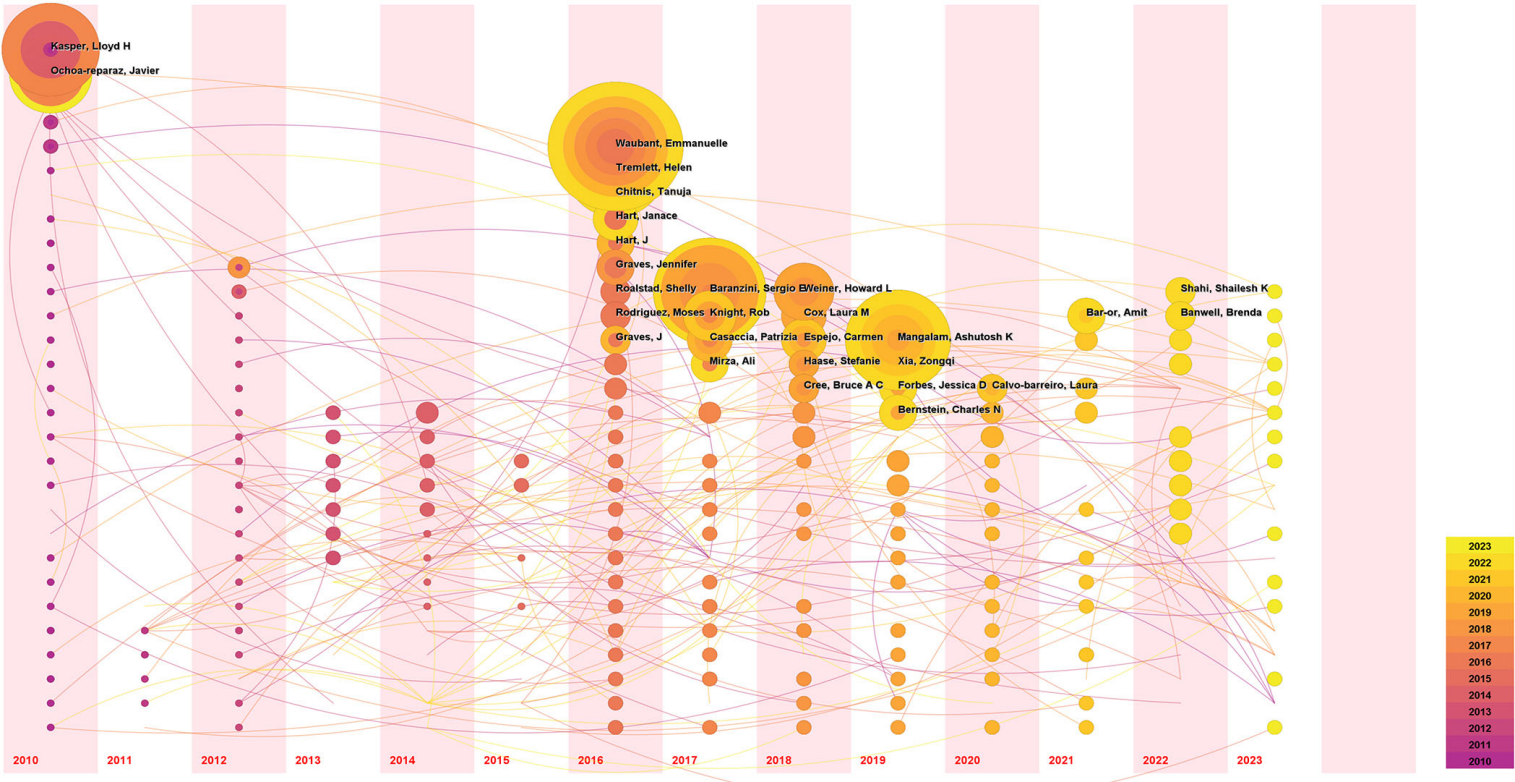
Visualization analysis of the temporal overlay cooperative network of research authors.

The author’s time nodes of publication were further analyzed utilizing the Temporal Map of CiteSpace, and the results are presented in **Figure 6**. Each circle represents an author, with the size of the circle proportional to the number of papers authored by the individual. The temporal zone of the circle indicates the year of the author’s first publication. Subsequent publication years are represented inside the circle in corresponding colors, forming an annual wheel where yellow denotes recent publications and purple represents earlier works. Lines connecting the circles denote collaborative relationships among authors. As depicted in the visualization, authors Kasper and Lloyd H. Ochoa-Reparaz are pioneers in the field of “GM and MS,” with research output dating back to 2010. Waubant and Emmanuelle made their debut in the field with publications in 2016, ranking second in publication volume and contributing continuously to the exploration of this domain, with recent findings being published.

### 3.5 Visual analysis of journals

**Figure 7:**
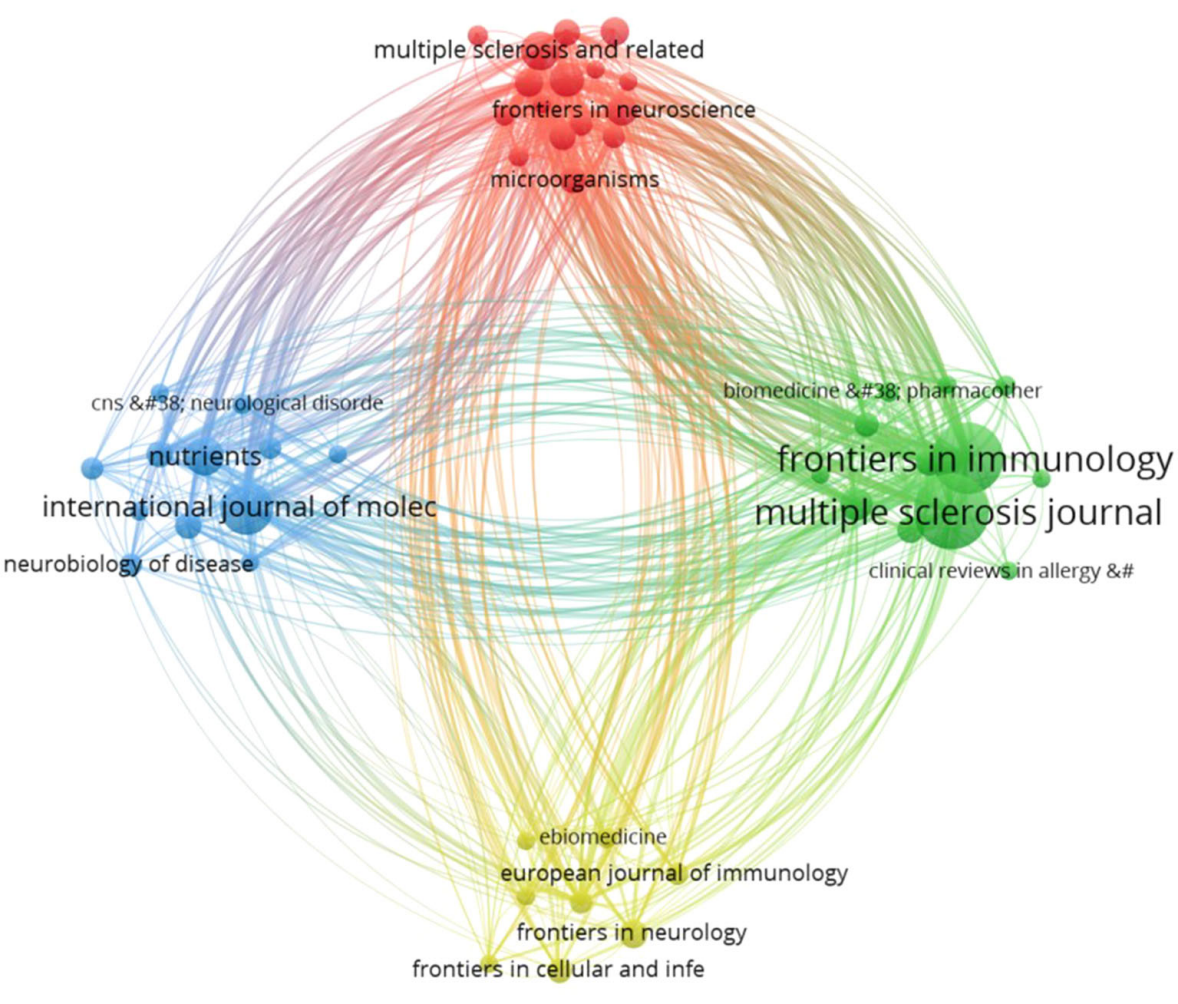
Visualization analysis of co-cited networks of journals.

A total of 359 journals have published articles in this particular field. By establishing a minimum requirement of 5 documents per journal, a total of 48 journals were selected, and a visualization representing these journals was obtained, showed in **Figure 7**. Each sphere symbolizes a journal, and its size is proportional to the number of documents issued by the respective journal. These spheres are grouped into clusters. The color represents distinct research direction clusters, categorized into four clusters. The thickness and density of the connecting lines indicate the strength of associations between the journals, with thicker lines suggesting stronger relationships. Among the top 5 journals based on publication volume, the first 4 journals are all in the JCR Q1 category with impact factors above 5. Specifically, Multiple Sclerosis Journal with 74 publications and an impact factor of 5.8; Frontiers in Immunology with 68 publications and an impact factor of 7.3; International Journal of Molecular Sciences with 36 publications and an impact factor of 5.6; Nutrients with 21 publications and an impact factor of 5.9. The 5th position is held by Multiple Sclerosis and Related Disorders, with an impact factor of 4.

**Figure 8:**
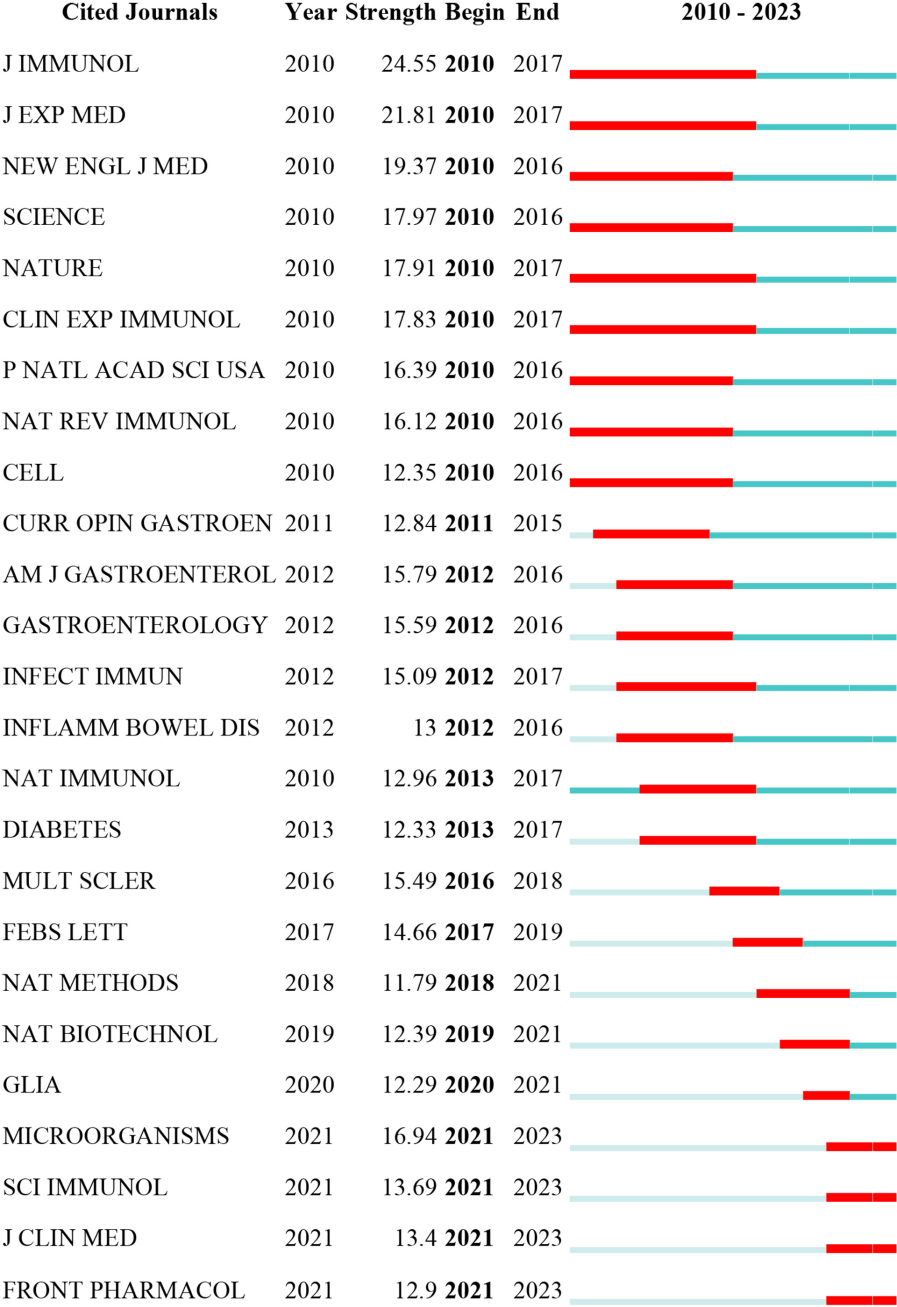
Top 25 cited journals with the strongest citation bursts.

**Figure 8** displays the top 25 cited journals with the strongest citation bursts. The red line indicates the period during which the journal experienced a citation burst. The top 5 strongest citation bursts periods occurred between 2010 and 2017, originating from the journals Journal of Immunology, J EXP MED, NEW ENGL J MED, SCIENCE, and NATURE.

### 3.6 Analysis of highly cited literature

We included TOP10 highly cited articles and TOP5 reviews, listed in **Tables 2**, **3**. among the top 10 highly cited articles, publication time was spread between 2015 and 2020, top 3 articles have been cited more than 500 times. Notably, the article by Jangi (2016) with the title ‘Alterations of the human gut microbiome in multiple sclerosis’ has the highest number of citations, reaching 796 times. In reviews, the most highly cited paper authored by Freedman in 2018 has amassed a total of 96 citations, uncovering potential mechanisms by which the GM can influence the development or mitigate the risk of MS.

**Table 2:** Analysis of the top 10 high cited research articles.

No.	Document	Title	Journal	DOI	Year	Number of citations
1	Jangi et al. (2016) (20)	Alterations of the human gut microbiome in multiple sclerosis	Nature Communications	10.1038/ncomms12015	2016	796
2	Chen et al. (2016) (11)	Multiple sclerosis patients have a distinct gut microbiota compared to healthy controls	Scientific Reports	10.1038/srep28484	2016	555
3	Berer et al. (2017) (21)	Gut microbiota from multiple sclerosis patients enables spontaneous autoimmune encephalomyelitis in mice	Proceedings of The National Academy of Sciences of The United States of America	10.1073/pnas.1711233114	2017	538
4	Miyake et al. (2015) (22)	Dysbiosis in the Gut Microbiota of Patients with Multiple Sclerosis, with a Striking Depletion of Species Belonging to Clostridia XIVa and IV Clusters	Plos One	10.1371/journal.pone.0137429	2015	477
5	Cantarel (2015)	Gut Microbiota in Multiple Sclerosis: Possible Influence of Immunomodulators	Journal of Investigative Medicine	10.1097/JIM.0000000000000192	2015	262
6	Tremlett (2016)	Gut microbiota in early pediatric multiple sclerosis: a case-control study	European Journal of Neurology	10.1111/ene.13026	2016	223
7	Tankou (2018)	A probiotic modulates the microbiome and immunity in multiple sclerosis	Annals of Neurology	10.1002/ana.25244	2018	133
8	Pröbstel (2020)	Gut microbiota-specific IgA+ B cells traffic to the CNS in active multiple sclerosis	Science Immunology	10.1126/sciimmunol.abc7191	2020	120
9	Saresella (2017)	Immunological and Clinical Effect of Diet Modulation of the Gut Microbiome in Multiple Sclerosis Patients: A Pilot Study	Frontiers in Immunology	10.3389/fimmu.2017.01391	2017	105
10	Swidsinski et al. (2017) (23)	Reduced Mass and Diversity of the Colonic Microbiome in Patients with Multiple Sclerosis and Their Improvement with Ketogenic Diet	Frontiers in Microbiology	10.3389/fmicb.2017.01141	2017	99

**Table 3:** Top 5 high cited review articles.

No.	Document	Title	Journal	DOI	Year	Number of citations
1	Freedman (2018) (24)	The “Gut Feeling”: Breaking Down the Role of Gut Microbiome in Multiple Sclerosis	Neurotherapeutics	10.1007/s13311-017-0588-x	2018	96
2	Riccio(2018)	Diet, Gut Microbiota, and Vitamins D plus A in Multiple Sclerosis	Neurotherapeutics	10.1007/s13311-017-0581-4	2018	95
3	Schepici (2019)	The Gut Microbiota in Multiple Sclerosis: An Overview of Clinical Trials	Cell Transplantation	10.1177/0963689719873890	2019	92
4	Chu et al. (2018) (25)	Gut Microbiota in Multiple Sclerosis and Experimental Autoimmune Encephalomyelitis: Current Applications and Future Perspectives	Mediators of Inflammation	10.1155/2018/8168717	2018	89
5	Bhargava (2014)	Gut Microbiome and Multiple Sclerosis	Current Neurology And Neuroscience Reports	10.1007/s11910-014-0492-2	2014	86

### 3.7 Analysis of keywords

**Figure 9:**
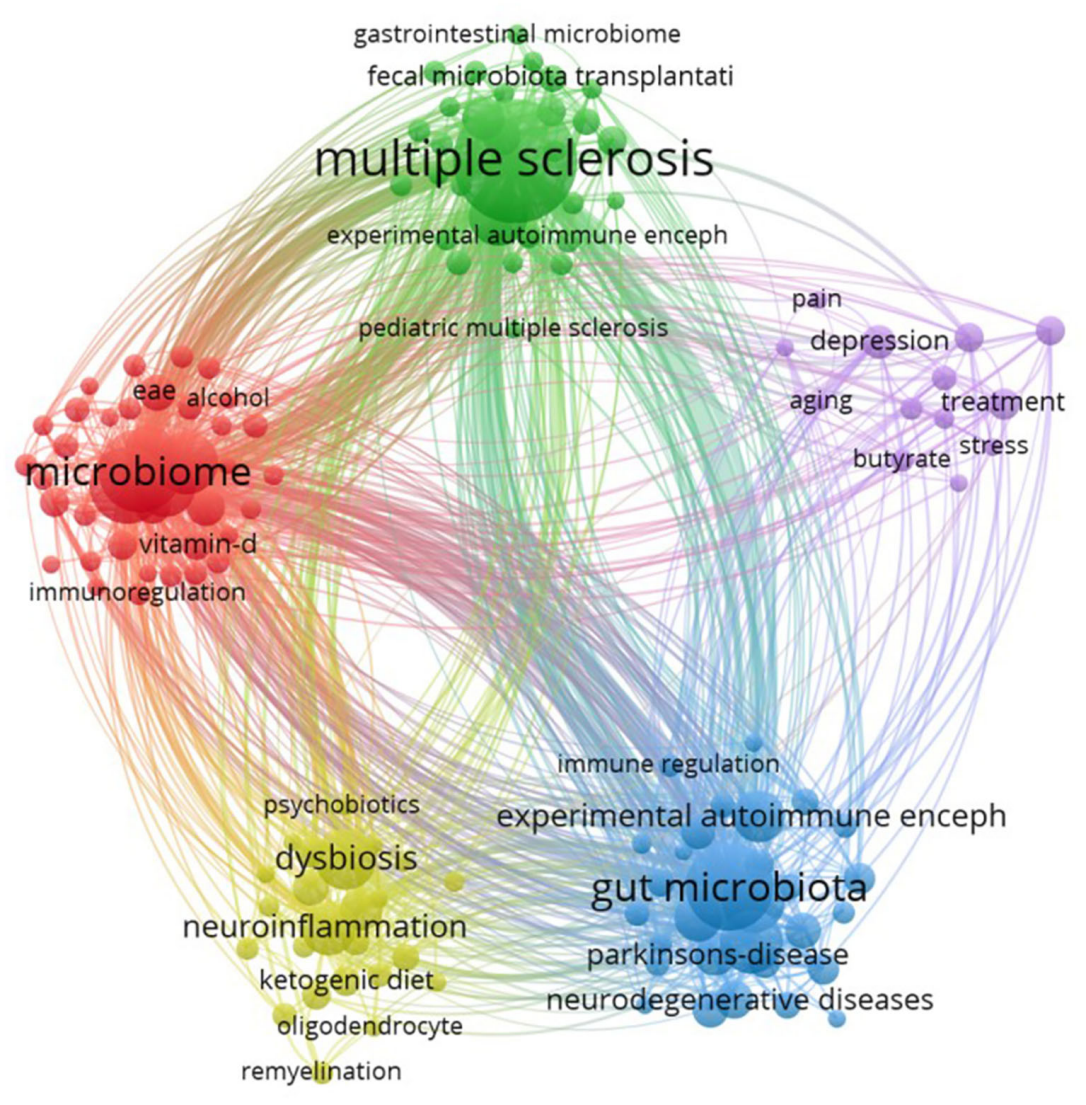
Visualization of the keywords co-occurrence network.

Analyzing keywords co-occurrence networks can help reveal the associations among keywords and understand the current research status of GM in MS field. We set the minimum occurrence frequency for each keyword to 5 times, and include 136 keywords in the visualization of the co-occurrence network (**Figure 9**). Every sphere stands for a keyword, the size proportional to the frequency of occurrence, the connected lines between spheres indicate relationships, and the color of sphere represents a different cluster, The literature keywords in this research field are categorized into five clusters, each representing an independent research direction.

The red cluster includes 52 keywords, with the top 10 weighted occurrences being “microbiome, probiotic microbiota, autoimmunity, diet, autoimmune diseases, vitamin D, amyotrophic lateral sclerosis, T-cells, cytokines, MS, therapy”, these keywords primarily center on the impact of microbiome and probiotics on autoimmunity, as well as the role of diet in autoimmune diseases. The green cluster comprises 30 keyword nodes, with “multiple sclerosis” and “gut microbiome” ranked as the top two, “inflammatory bowel disease” as the third, and “rheumatoid arthritis,” “intestinal permeability,” “systemic lupus erythematosus,” and “fecal microbiota transplantation” being high-frequency keywords. The cluster predominantly reflects research directions concerning the relationship between GM and systemic immune-mediated diseases, potentially offering insights into the treatment and intervention of related disorders. The blue cluster comprises 30 keyword nodes, primarily concentrated on investigating the association and mechanisms between neurological diseases and GM. The high-frequency keywords in this cluster include inflammation, gut-brain axis, experimental autoimmune encephalomyelitis, Alzheimer’s disease, Parkinson’s disease, neurodegenerative diseases, immune system, oxidative stress, and short-chain fatty acids. Yellow cluster comprises 24 keyword nodes, encompassing representative keywords such as dysbiosis, neuroinflammation, neurodegeneration, immunity, ketogenic diet, and microglia. This suggests that the primary focus of this cluster is the association between dysbiosis and neurologic disorders. The purple cluster comprised 30 key nodes, primarily focused on the field of mental health, particularly concerning depression (including depression, treatment, gut dysbiosis, immunomodulation, and melatonin).

**Figure 10:**
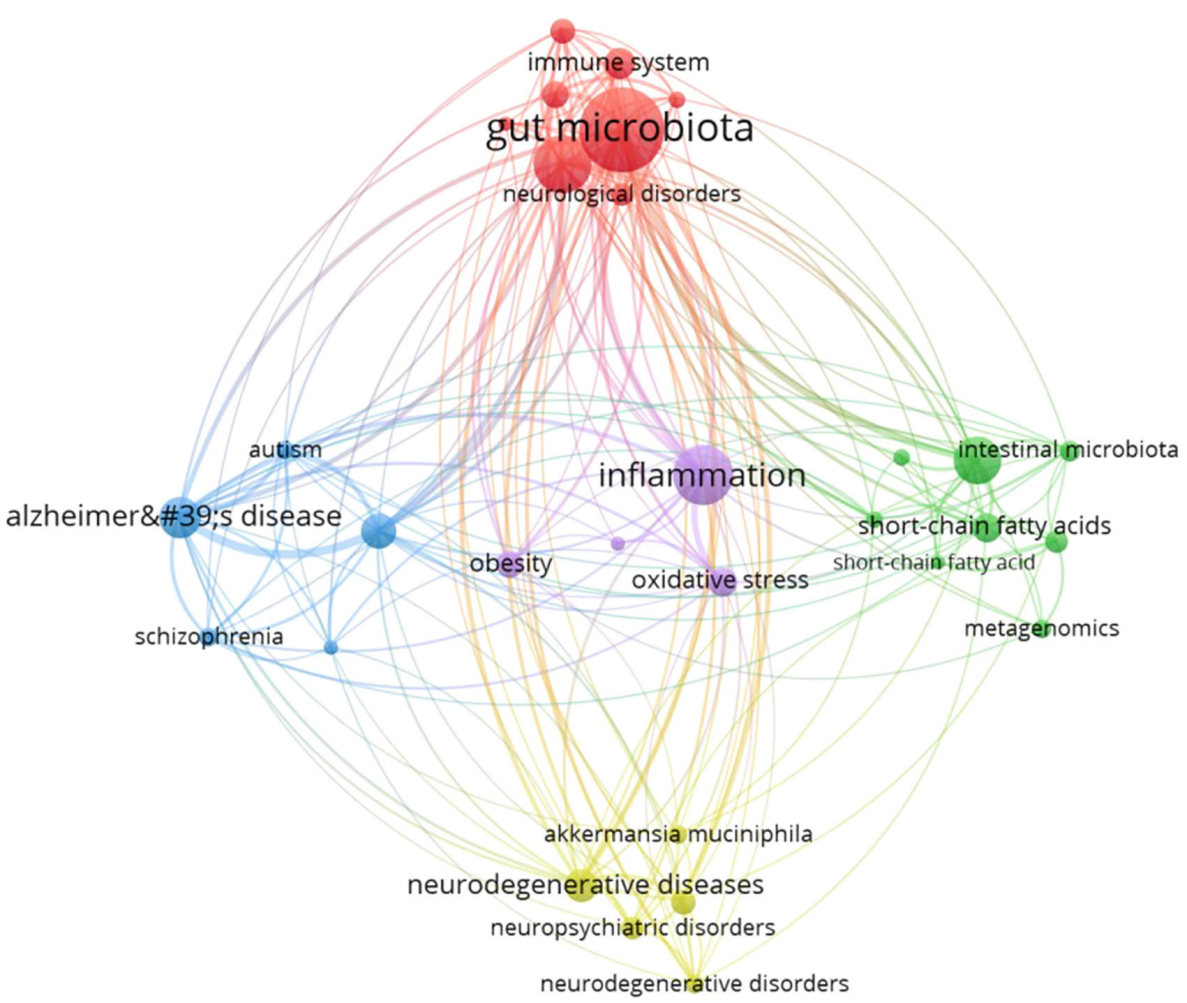
Subcluster analysis based on gut microbiota.

In this study, GM was subjected to sub-cluster analysis based on all the extracted literature (**Figure 10**). The analysis revealed a substantial body of research linking GM to neuropsychiatric disorders such as schizophrenia and autism, as well as neurodegenerative diseases like Alzheimer’s disease. The molecular metabolic mechanisms including short-chain fatty acids, metagenomics, oxidative stress, and inflammation, are currently at the forefront of research.

### 3.8 Evolution of keywords and research trends

**Figure 11:**
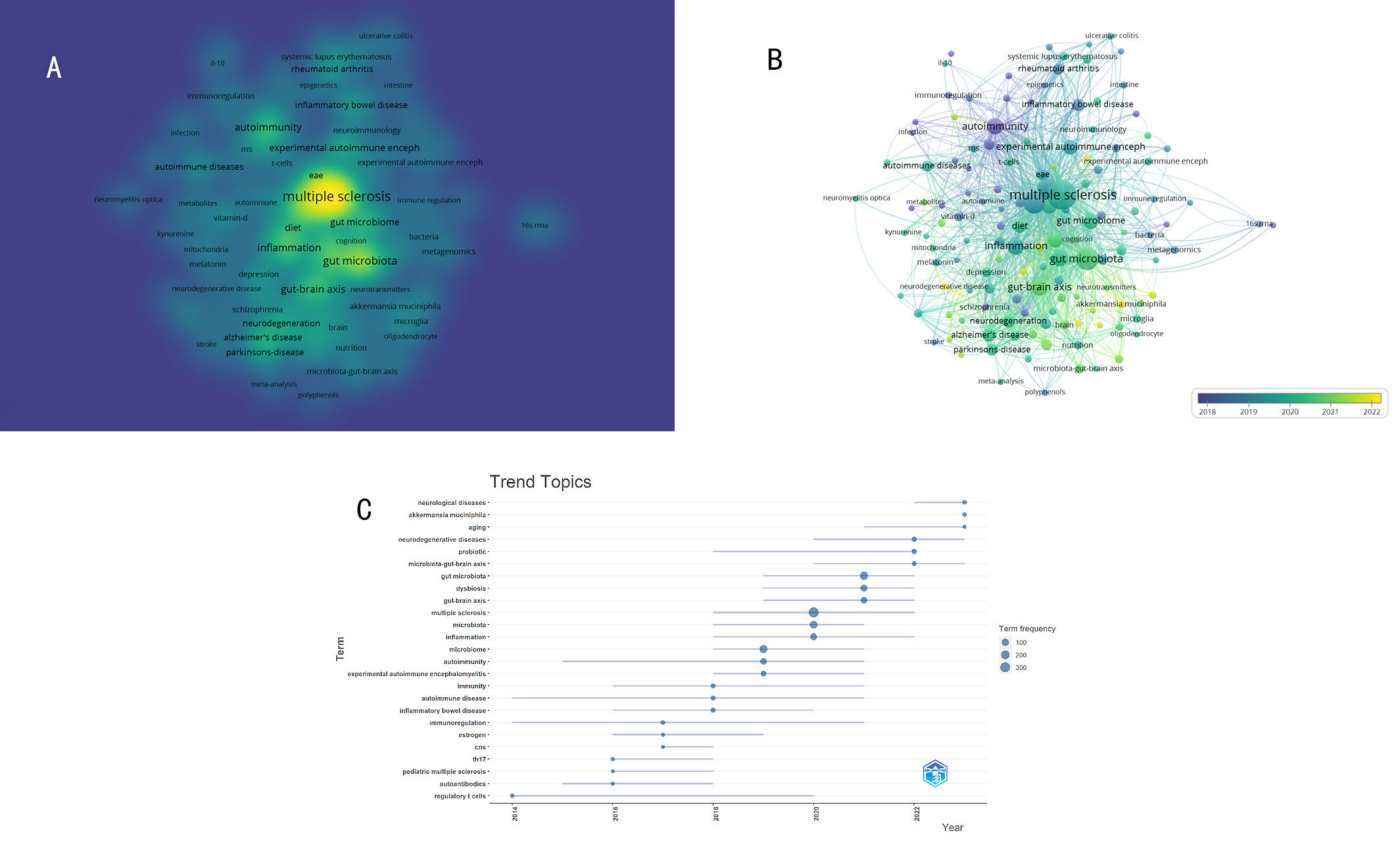
**(A)** The density visualization of the keywords co-occurrence network. **(B)** Visualization analysis of timing overlay in the keyword co-occurrence network. **(C)** Visualization analysis of the historical evolution of keywords.

The density visualization of the keywords co-occurrence network was shown in **Figure 11A**. The figure clearly displayed the current research landscape centered around the topics of “MS” and “GM”. The 16S rRNA gene sequencing technology serves as a crucial tool for analyzing the structure of the GM. Experimental autoimmune encephalomyelitis (EAE) stands as the most common mouse model in simulating MS. The GM is associated not only with various autoimmune diseases such as inflammatory bowel disease, rheumatoid arthritis, systemic lupus erythematosus, and optic neuritis but also with neurodegenerative diseases like Parkinson’s disease, Alzheimer’s disease, and other psychiatric disorders(schizophrenia, depression). The involvement or regulation of these diseases’ mechanisms by the GM is linked to multiple factors including diet, inflammation, T-cells, autoimmunity, and metabolites. These keywords underscore the intimate interplay between GM and host immunity and the nervous system, suggesting a critical role in the pathogenesis and progression of various diseases.

The average year of keyword emergence reflects the evolution of keywords. **Figure 11B** depicts the visualization of keyword timing, each node represents a keyword and is color-coded based on its average year of appearance. The most frequently occurring keywords appear predominantly after 2018, indicating GM in the MS field is an emerging research hotspot. Among the early keywords are inflammatory bowel disease, rheumatoid arthritis, autoimmunity, and immunoregulation, after 2020, keywords such as neurodegenerative diseases, schizophrenia, depression, and the gut-brain axis have emerged, suggesting a shift in focus towards the relationship between the GM and neuropsychiatric disorders.

Analysis of “Trend Topics” provides a more intuitive insight into the historical evolution of keywords in the field. As shown in **Figure 11C**, prior to 2018, the prominent keywords included immunity, autoimmune disease, inflammatory bowel disease, estrogen, and Th17, indicating many research focuses on the role of GM in the immune mechanism of MS. From 2019 to 2020, high-frequency keywords evolved to include “EAE, microbiome, inflammation, MS”, indicating a significant focus on the role of GM in animal models and clinical studies of MS. Post 2021, the emergence of high-frequency keywords such as “gut-brain axis, dysbiosis, probiotic, neurodegenerative disease, aging” suggests a shift in researchers’ interest and emphasis towards understanding the impact of GM in neurodegenerative diseases and the aging process.

## 4 Discussion

### 4.1 Research overview of the GM in the field of MS

From 2010 to 2023, a total of 1,019 records were extracted on the field of GM and MS from the Web of Science database. As shown in **Figure 2**, a continuous upward trend in publication volume and annual citations over the years. Although in the period from 2010 to 2012, the annual publication output was only in single digits, totaling 15 articles, accounting for 1.4% of the total output during the period of 2010-2023. Subsequently, there has been a substantial increase each year with at least 10 publications, with the publications in the last three years (447) accounting for 44% of the total output. The increasing number of publications since 2013 suggests that the study of GM in MS is becoming a prominent and valuable research area of high significance. This progress may be attributed to the release of the second phase of the Integrative Human Microbiome Project (iHMP) funded by the National Institutes of Health in 2013 (26). The emergence of new research tools, such as 16S rRNA gene pyrosequencing, and metagenomic shotgun sequencing further propelled advancements in the field of GM research. These methods provide researchers with powerful tools to delve deeper into the complexities of the microbial communities residing in the gut.

The United States has the most significant influence in the field of GM and MS, with the highest publication volume at 393 articles (37.90%), surpassing Italy in second place with 114 articles (10.99%). At the same time, it serves as the primary facilitator of collaboration intensity, fostering cooperative partnerships in this field with countries such as Canada, Germany, Italy, the United Kingdom, and Spain. While a total of 1581 institutions are involved in the field of GM and MS, only 33 institutions have published more than 10 articles, indicating that the majority of these institutions have not conducted a thorough investigation into the field. As shown in **Figure 4**, two institutions from the United States, The University of California, San Francisco, and Harvard Medical School have solidified their positions as major contributors in this field, leading the list with the highest number of publications. The University of California, San Francisco is distinguished by its centrality and proactive approach to collaboration with other institutions.

The total number of publications and citations of research authors are important indicators for evaluating their academic contributions and influence. Kasper, Lloyd h, and Ochoa-reparaz, Javier from USA, have had the most significant influence in this field, as evidenced by their most publications and citations. Noteworthy, Emmanuelle Waubant, an author affiliated with the University of California, San Francisco, is ranked second in publication output and third in citations, with publication in the field in 2016, and has been continuously and actively exploring this domain (**Figure 6**). This indicates that the author may be the leading and most influential figure in this field in the future. Analysis of journal publications revealed that 359 journals were engaged in the field of GM and MS. Notably, the four leading journals with impact factors exceeding 5 demonstrate the high quality and academic significance of research in this area.

Analysis of highly cited literature can help researchers quickly discover research results that have significant influence. The most cited original research study was conducted by Jangi in 2016 (20), titled “Alterations of the human gut microbiome in multiple sclerosis”. In this study, the author examined the GM of 60 patients with MS and 43 healthy controls using 16S rRNA sequencing. The results showed that MS patients had lower levels of Butyricimonas and higher amounts of Methanobrevibacter and Akkermansia. Variations in dendritic cell maturation, interferon signaling, and NF-kB signaling pathways were linked to these alterations in the gene expression of circulating T cells and monocytes. Also in 2016, Chen et al (11). published a paper titled “Multiple sclerosis patients have a distinct gut microbiota compared to healthy controls” which ranks second in total citations. This study employed hypervariable tag sequencing of the 16S ribosomal RNA gene to investigate the microbial community profile, the results showed higher levels of Pseudomonas, Mycoplana, Haemophilus, Blautia, and Dorea genera in MS patients, while the control group demonstrated elevated levels of Parabacteroides, Adlercreutzia, and Prevotella genera. MS patients exhibit distinct microbial community characteristics. The study titled “Gut microbiota from multiple sclerosis patients enables spontaneous autoimmune encephalomyelitis in mice” by Berber (21), published in 2017, ranks third in total citations. The study reports that when microbiota derived from MS twins were transplanted into a spontaneous autoimmune encephalomyelitis transgenic mouse model, the occurrence of autoimmunity was significantly higher compared to microbiota from healthy twin donors. These results suggest that the microbiota from individuals with MS may harbor factors that trigger an MS-like autoimmune response in an experimental mouse model. These studies laid the groundwork for understanding the role of the GM in the field of MS by elucidating the specific microbial changes associated with the disease and their potential impact on immune processes. The most cited review article by Freedman in 2018 (24), titled “The ‘Gut Feeling’: Breaking Down the Role of Gut Microbiome in Multiple Sclerosis” systematically reviewed the results of different studies on changes in the microbiome of MS, analyzed in-depth the potential mechanisms through which the GM may either trigger or prevent MS, paving the way for the development of therapies based on GM to treat MS. An in-depth analysis of the highly cited literature enables us to develop a thorough understanding of the evidence linked GM and MS, offering valuable insights into this specific field.

### 4.2 Hot spots evolution and future trends in GM and MS

Keywords typically embody crucial concepts, topics, or methodologies within an article, serving as a quick means for readers to grasp the primary content swiftly. The frequency of keywords appearing in literature in a field can reflect the research hotspots of that field. Analysis of keyword co-occurrence can elucidate the current research status in the field while visualizing keyword timing can illustrate the evolution of research hotspots and potential future directions. In our study, by analyzing the keyword co-occurrence network (**Figure 9**), we identified five distinct research directions related to GM in the context of MS. These directions encompass the influence of the microbiome and probiotics on autoimmunity, the correlation between GM composition and systemic immune-mediated diseases, the connections and mechanisms associating neurological disorders with GM, and the implications of GM dysbiosis for neurological and psychiatric disease. The visualization of keyword timing (**Figure 11B**) and the analysis of ‘Trend Topics’ (**Figure 11C**) reveal that the influence of GM in the field of MS has progressively extended to neurodegenerative diseases and psychiatric disorders such as autism spectrum disorder in recent years, particularly after 2020, this indicates the future exploration direction of GM. Keywords like the ketogenic diet, probiotics, and fecal microbiota transplantation not only embody theoretical advancements but also reflect the practical application of GM-based therapies in clinical research. Keywords with higher weights and an average appearance after 2018 in molecular mechanisms, including tryptophan, butyrate, short-chain fatty acids, neuroinflammation, T-cells, microglia, melatonin, oxidative stress, and vitamin D, emphasize the focal points in the exploration of GM mechanisms in MS field.

### 4.3 The change of GM in MS

Numerous studies have identified evidence of alterations in the composition of microbiota in MS patients (27–30). Sushrut Jangi et al (26) identified higher levels of Methanobrevibacter and Akkermansia, and decreased levels of Butyricimonas in 60 MS patients using 16S rRNA sequencing. Furthermore, MS patients undergoing disease-modifying treatments exhibited higher abundances of Prevotella and Sutterella, while lower levels of Sarcina were observed in comparison to untreated patients (20). A study conducted by Cox LM reported an increase in the abundance of Clostridium bolteae, Ruthenibacterium lactatiformans, and Akkermansia, along with a decrease in Blautia wexlerae, Dorea formicigenerans, and Erysipelotrichaceae CCMM, in both progressive MS and relapsing-remitting MS(RRMS)compared to healthy controls (10). Furthermore, elevated levels of Enterobacteriaceae and Clostridium g24 FCEY, coupled with decreased levels of Blautia and Agathobaculum, were specific to progressive MS (10). Florence Thirion et al. investigated fecal microbial DNA in a cohort of 148 MS patients, found that 61 bacterial species exhibited varying abundance levels in MS patients compared to healthy controls (14). A study that analyzed the microbiota isolated from the small intestinal tissues of MS patients showed higher ratios of Firmicutes/Bacteroidetes, higher relative abundances of Streptococcus, and lower presences of Prevotella strains when compared to the healthy group (31). As part of the International Multiple Sclerosis Microbiome Study (iMSMS), the GM of 576 MS patients and 1152 healthy controls were analyzed. The results of the study showed that while the abundance of Akkermansia muciniphila, Ruthenibacterium lactatiformans, Hungatella hathewayi, and Eisenbergiella tayi were much more prevalent in MS patients, Faecalibacterium prausnitzii and other Blautia species were less common (13). The discrepancies among studies in microbial composition may be associated with detection methods, sample inclusion, geographical locations, and dietary habits, among others.

Evidence from animal study suggests that microbiota transplantation from patients with MS into germ-free(GF) mice validated exacerbated symptoms in experimental autoimmune encephalomyelitis (EAE) mice and a decreased ratio of IL-10+ regulatory T cells compared to controls (32). Another study corroborated this finding, indicating that fecal transplants from MS patients are more likely to trigger EAE in GF mice than transplants from healthy control subjects (21). Additionally, mice raised in GF environments exhibit a notably milder form of EAE in contrast to those raised under conventional conditions exposed to microorganisms. On the contrary, colonization of GF mice with segmented filamentous bacteria led to an increase in IL-17 levels in the gut, which facilitated the proliferation of Th17 cells and ultimately triggered the onset of EAE (33). These findings underscore the influence of microbiota on EAE, suggesting that targeting microbiota intervention could be a viable approach for treating MS (18). One particular study found that FMT altered the gut microbiome composition in EAE mice (33). Specifically, FMT increased the relative abundance of the bacterial phyla Firmicutes and Proteobacteria, while decreasing the abundance of Bacteroides and Actinobacteria, compared to the control EAE group. Importantly, the clinical scores (a measure of disease severity) were significantly lower in the EAE mice that received FMT, compared to the control EAE group (34). Besides that, other studies have identified specific probiotic bacteria, such as strains of Lactobacillus and Bifidobacterium, that have shown potential to alleviate symptoms in the EAE model (35–37). Moreover, both interventions with Clostridium butyricum and norfloxacin targeting GM have been documented to alleviate symptoms of EAE mice through the modulation of Th17 and Treg immune responses (38). Similarly, vancomycin treatment in mice with EAE improved symptoms by modulating the gut-brain axis through the microbiota, promoting the growth of specific bacterial species known to stimulate the formation of regulatory T cells (39). Notably, two studies suggested that treatment with the human commensal, bacterium Prevotella histocola is as effective as the MS therapies interferon beta and glatiramer acetate therapies in the EAE model (40, 41). These data from experimental and clinical studies indicate the potential significance of the microbiota-gut-brain axis in the pathogenesis of MS.

### 4.4 The pathogenic mechanism of GM dysbiosis involved in MS

Even though the precise mechanism by which GM is involved in the pathogenesis of MS has not been fully elucidated, various review articles discuss the potential role of GM in MS from different perspectives. Microbiota-gut-brain axis refers to a complex bidirectional communication between the gastrointestinal microbiome and the central nervous system (CNS), involving the enteric nervous system (ENS), CNS, immune system, autonomic nervous system, and GM (8). CNS can be affected by the GM via neuroendocrine, neurotransmitter, and neuroimmune signaling pathways (42). The influence of GM on the physiological properties of the ENS has been highlighted in a recent review by Zhang, recognizing it as an additional contributing factor in MS (28). This link is reinforced by research employing EAE models (43, 44). Additionally, the GM influences immune cells located in the gastric mucosa (45, 46), leading to increased proinflammatory cytokines and contributing to the reduced permeability of the blood-brain barrier (BBB). This alteration allows immune cells and cytokines from the peripheral circulation to breach the brain, ultimately triggering chronic neuroinflammation (47). Furthermore, GM can influence metabolism and the production of butyrate (48), which may lead to deficits in mitochondrial function and antioxidant capacity (49), serving as a significant driver in the pathological changes associated with MS (49–51). The review underscores the crucial role of Polyunsaturated Fatty Acids (PUFAs) as metabolites of GM. Preliminary research suggests that PUFAs have the potential to inhibit phospholipase activity, decrease the production of inflammatory mediators, and lower levels of arachidonic acid (52, 53). The observed disease-modifying effects and reduced incidence rates in EAE animal models and clinical studies highlight the therapeutic promise of high-dose PUFAs (54–58). A review article by Samantha N focused on the influence of gut bacteria metabolites, such as short-chain fatty acids (SCFAs), bile acids, phytoestrogens, tryptophan, and choline, on host immune responses and their role in MS (24). Among these, SCFAs have been extensively studied in the context of host immunity (59–61), altering immune-defensive of the intestinal epithelial cells (IECs) (62), leukocyte phenotype, and/or effector function in the gut, affecting immune responses in local or systemic pathophysiological situations (62–65). Bile acid metabolism is implicated in the onset and progression of MS through various pathways, which include the regulation of immune cell activation and function, the influence on the composition and functionality of the intestinal microbiota, as well as the modulation of intestinal barrier integrity and inflammatory responses. GM can transform phytoestrogens into compounds with anti-inflammatory properties, thus alleviating the symptoms of MS by regulating the anti-inflammatory effects of the immune response (24). Tryptophan can be metabolized by specific gut bacteria into indole and its derivatives, which can enhance intestinal barrier function and modulate the activity of immune cells (66–68). Other review articles expounded on the evidence of GM in the onset, progression, and intervention of MS from both clinical and EAE animal model perspectives. The mechanisms elucidated in these studies primarily focus on the influence of immunoregulatory molecules including IFN-γ and IL-17, Th1 and Th17 cells, and interleukin-10 (IL-10) (9, 25, 27, 69).

Collectively, GM dysbiosis in both clinical and experimental studies of MS is well-established. Specifically, MS patients show a higher abundance of Akkermansia muciniphila, Blautia, Methanobrevibacter, Pseudomonas, Bifidobacterium, Desulfovibrionaceae, and others compared to healthy controls. Conversely, lower levels of Faecalibacterium prausnitzii, Parabacteroides, Prevotella, Lachnospiraceae, Ruminococcaceae, Bacteroides, Faecalibacterium, and Adlercreutzia are observed and presented in the **Table 4**. These alterations in the GM can influence the pathophysiology of MS through various mechanisms, either directly or via their metabolites.

**Table 4:** Bacterial composition alterations in MS.

Bacteria	Trend	Reference	Bacteria	Trend	Reference
Akkermansia muciniphila	↑	(20) (32) (21) (28)	Faecalibacterium prausnitzii	↓	(22) (28) (23)
Blautia	↑	(11) (28)	Parabacteroides	↓	(11, 13, 28)
Methanobrevibacter	↑	(20, 28)	Prevotella	↓	(11, 32, 70)
Pseudomonas	↑	(11) (28)	Lachnospiraceae	↓	(28)
Haemophilus	↑	(20, 28)	Ruminococcaceae	↓	(28, 70)
Eggerthella lenta	↑	(22, 32)	Faecalibacterium	↓	(11, 28)
Bifidobacterium	↑	(28, 70)			
Desulfovibrionaceae	↑	(20, 70)			
Acinetobacter calcoaceticus	↑	(13, 27)			

The symbol ↑ represents an increase, while the symbol ↓ represents a decrease.

### 4.5 Clinical applications based on the treatment of GM dysbiosis

Given the pivotal role of the GM in MS pathology through its influence on the immune response and metabolic pathways, there is growing research interest in investigating the potential therapeutic benefits of targeting the GM for the management of MS. Various interventions such as dietary interventions, FMT, pharmacological interventions are being explored in this context (18, 28, 71). Dietary interventions involve the modification of dietary components and regimens to influence the composition of the GM or indirectly impact the host immune system. Dietary components include vitamin D, vitamin A, salt, polyphenols, thiol compounds, fibers, and SCFAs (72). Dietary regimens consist of a low-fat diet, caloric restriction, ketogenic diet (low-carbohydrate, high-fat diet), plant-based diets (vegetarian and vegan diets), and the Mediterranean diet (enriched with fruits, vegetables, whole grains, and unsaturated fats, while limited meat consumption) (72), While these dietary protocols have demonstrated effectiveness in alleviating disease symptoms, reducing relapse rates, and slowing disease progression in both clinical and preclinical research models of MS (73–77), conflicted findings also existed (78–80), emphasizing the need for cautious consideration in their practical application. FMT involves the transfer of fecal matter from a healthy donor into a patient after the administration of high-dose broad-spectrum antibiotics. This procedure aims to restore microbial equilibrium and has been proven as an effective approach for inflammatory bowel syndrome and systemic autoimmune diseases (81, 82). FMT has been observed to delay disease onset and alleviate clinical symptoms by modifying gut flora composition, promoting SCFA production, preventing relapses, and increasing brain-derived neurotrophic factor levels in animal models (83). Clinical cases have also reported positive outcomes with FMT in MS patients (84, 85), further supporting its potential as a therapeutic intervention. Pharmacological interventions use three methods: antibiotic use, supplementation of probiotics and prebiotics, and immunomodulatory drugs to alter the composition of the GM, improve gut microbiome balance, and influence the immune response of the GM and the host. Oral antibiotics have shown promise in delaying the onset of EAE in animal models (86, 87). In particular, minocycline demonstrated significant efficacy in alleviating symptoms and impeding disease progression in a randomized, controlled clinical trial (88). Supplementation with prebiotics is widely recognized for its role in managing gut dysbiosis-related intestinal disorders. Probiotics have been shown to exert beneficial effects through various mechanisms, such as producing antimicrobial compounds, modulating the activity of immune cells, and supporting the integrity of the gut barrier, as evidenced by studies conducted *in vitro* and animal models (89, 90). Several animal studies have provided evidence supporting the effectiveness of probiotics administration in managing EAE (35, 91–93), including reducing the incidence, delaying symptom onset, and alleviating symptoms. A meta-analysis also demonstrated improvements in depression and limb weakness in patients with RRMS (94). Moreover, certain medications, such as Fingolimod and teriflunomide, have been reported to potentially suppress the growth of neurotoxin-producing gut bacteria. It can be seen that researchers are actively investigating the utilization of GM in the management of MS from various angles. However, further investigation is warranted to understand the mechanisms by which these approaches operate within the intrinsic regulation of EAE or MS patients.

### 4.6 Conclusion

Through a scientometric analysis of the GM in MS based on data from the WOSCC, this study has provided a systematic and comprehensive understanding of the historical progression and current status of research on the GM in MS. This field has captured considerable attention from researchers worldwide, and continues to evolve. The scholarly output in this area has notably increased over the past decade, with established literature affirming a direct association between the GM and MS. Future research trends should focus on emphasizing the role of GM dysbiosis in the pathogenesis of MS, investigating specific microbial taxa and metabolites with potential protective or pathogenic roles, and examining microbiome-targeted interventions as innovative therapeutic strategies. While various interventions targeting the GM have been developed to enhance outcomes in EAE or MS, the underlying interaction mechanisms between GM and MS remain insufficiently understood. Additionally, the absence of large-scale, randomized controlled clinical trials further highlights the necessity for a comprehensive assessment of the efficacy and drawbacks of such interventions. These unresolved issues may represent potential crucial areas for future research to address. In conclusion, this study offers valuable insights by providing an overall overview of research and highlighting significant and innovative works in the field. It enhances our comprehension of the GM in MS and serves as a crucial reference for prospective studies.

## Data Availability

The original contributions presented in the study are included in the article/supplementary material. Further inquiries can be directed to the corresponding author.

## References

[1] Jakimovski D Bittner S Zivadinov R Morrow SA Benedict RH Zipp F. Multiple sclerosis. Lancet. (2024) 403:183–202. doi: 10.1016/S0140-6736(23)01473-3 37949093

[2] Filippi M Bar-Or A Piehl F Preziosa P Solari A Vukusic S. Multiple sclerosis. Nat Rev Dis Primers. (2018) 4:43. doi: 10.1038/s41572-018-0041-4 30410033

[3] Cree B Oksenberg JR Hauser SL. Multiple sclerosis: two decades of progress. Lancet Neurol. (2022) 21:211–4. doi: 10.1016/S1474-4422(22)00040-0 PMC8893310 35182500

[4] Qin J Li R Raes J Arumugam M Burgdorf KS Manichanh C. A human gut microbial gene catalogue established by metagenomic sequencing. Nature. (2010) 464:59–65. doi: 10.1038/nature08821 20203603 PMC3779803

[5] Thursby E Juge N. Introduction to the human gut microbiota. Biochem J. (2017) 474:1823–36. doi: 10.1042/BCJ20160510 PMC5433529 28512250

[6] Dominguez-Bello MG Blaser MJ Ley RE Knight R. Development of the human gastrointestinal microbiota and insights from high-throughput sequencing. Gastroenterology. (2011) 140:1713–9. doi: 10.1053/j.gastro.2011.02.011 PMC10924805 21530737

[7] Rinninella E Raoul P Cintoni M Franceschi F Miggiano G Gasbarrini A. What is the healthy gut microbiota composition? A changing ecosystem across age, environment, diet, and diseases. Microorganisms. (2019) 7. doi: 10.3390/microorganisms7010014 PMC6351938 30634578

[8] Cryan JF O’Riordan KJ Cowan C Sandhu KV Bastiaanssen T Boehme M. The microbiota-gut-brain axis. Physiol Rev. (2019) 99:1877–2013. doi: 10.1152/physrev.00018.2018 31460832

[9] Sittipo P Choi J Lee S Lee YK. The function of gut microbiota in immune-related neurological disorders: a review. J Neuroinflamm. (2022) 19:154. doi: 10.1186/s12974-022-02510-1 PMC9199126 35706008

[10] Cox LM Maghzi AH Liu S Tankou SK Dhang FH Willocq V. Gut microbiome in progressive multiple sclerosis. Ann Neurol. (2021) 89:1195–211. doi: 10.1002/ana.26084 PMC8132291 33876477

[11] Chen J Chia N Kalari KR Yao JZ Novotna M Paz SM. Multiple sclerosis patients have a distinct gut microbiota compared to healthy controls. Sci Rep. (2016) 6:28484. doi: 10.1038/srep28484 27346372 PMC4921909

[12] Takewaki D Suda W Sato W Takayasu L Kumar N Kimura K. Alterations of the gut ecological and functional microenvironment in different stages of multiple sclerosis. Proc Natl Acad Sci USA. (2020) 117:22402–12. doi: 10.1073/pnas.2011703117 PMC7486801 32839304

[13] Consortium I. Gut microbiome of multiple sclerosis patients and paired household healthy controls reveal associations with disease risk and course. Cell. (2022) 185:3467–86. doi: 10.1016/j.cell.2022.08.021 PMC10143502 36113426

[14] Thirion F Sellebjerg F Fan Y Lyu L Hansen TH Pons N. The gut microbiota in multiple sclerosis varies with disease activity. Genome Med. (2023) 15:1. doi: 10.1186/s13073-022-01148-1 36604748 PMC9814178

[15] Bibolini MJ Chanaday NL Baez NS Degano AL Monferran CG Roth GA. Inhibitory role of diazepam on autoimmune inflammation in rats with experimental autoimmune encephalomyelitis. Neuroscience. (2011) 199:421–8. doi: 10.1016/j.neuroscience.2011.08.076 21964471

[16] Wang Y Telesford KM Ochoa-Reparaz J Haque-Begum S Christy M Kasper EJ. An intestinal commensal symbiosis factor controls neuroinflammation via TLR2-mediated CD39 signalling. Nat Commun. (2014) 5:4432. doi: 10.1038/ncomms5432 25043484 PMC4118494

[17] Haghikia A Jörg S Duscha A Berg J Manzel A Waschbisch A. Dietary fatty acids directly impact central nervous system autoimmunity via the small intestine. Immunity. (2015) 43:817–29. doi: 10.1016/j.immuni.2015.09.007 26488817

[18] Kujawa D Laczmanski L Budrewicz S Pokryszko-Dragan A Podbielska M. Targeting gut microbiota: new therapeutic opportunities in multiple sclerosis. Gut Microbes. (2023) 15:2274126. doi: 10.1080/19490976.2023.2274126 37979154 PMC10730225

[19] Stidham RW Sauder K Higgins P. Using bibliometrics to advance your academic career. Gastroenterology. (2012) 143:520–3. doi: 10.1053/j.gastro.2012.07.024 22828355

[20] Jangi S Gandhi R Cox LM Li N von Glehn F Yan R. Alterations of the human gut microbiome in multiple sclerosis. Nat Commun. (2016) 7:12015. doi: 10.1038/ncomms12015 27352007 PMC4931233

[21] Berer K Gerdes LA Cekanaviciute E Jia X Xiao L Xia Z. Gut microbiota from multiple sclerosis patients enables spontaneous autoimmune encephalomyelitis in mice. Proc Natl Acad Sci USA. (2017) 114:10719–24. doi: 10.1073/pnas.1711233114 PMC5635914 28893994

[22] Miyake S Kim S Suda W Oshima K Nakamura M Matsuoka T. Dysbiosis in the gut microbiota of patients with multiple sclerosis, with a striking depletion of species belonging to clostridia XIVa and IV clusters. PloS One. (2015) 10:e137429. doi: 10.1371/journal.pone.0137429 PMC4569432 26367776

[23] Swidsinski A Dorffel Y Loening-Baucke V Gille C Goktas O Reisshauer A. Reduced mass and diversity of the colonic microbiome in patients with multiple sclerosis and their improvement with ketogenic diet. Front Microbiol. (2017) 8:1141. doi: 10.3389/fmicb.2017.01141 28702003 PMC5488402

[24] Freedman SN Shahi SK Mangalam AK. The “Gut feeling”: breaking down the role of gut microbiome in multiple sclerosis. Neurotherapeutics. (2018) 15:109–25. doi: 10.1007/s13311-017-0588-x PMC5794701 29204955

[25] Chu F Shi M Lang Y Shen D Jin T Zhu J. Gut microbiota in multiple sclerosis and experimental autoimmune encephalomyelitis: current applications and future perspectives. Mediators Inflamm. (2018) 2018:1–17. doi: 10.1155/2018/8168717 PMC5902007 29805314

[26] Integrative HMP (iHMP) Research Network Consortium. The Integrative Human Microbiome Project: dynamic analysis of microbiome-host omics profiles during periods of human health and disease. Cell Host Microbe. (2014) 16:276–89. doi: 10.1016/j.chom.2014.08.014 PMC5109542 25211071

[27] Altieri C Speranza B Corbo MR Sinigaglia M Bevilacqua A. Gut-microbiota, and multiple sclerosis: background, evidence, and perspectives. Nutrients. (2023) 15:942. doi: 10.3390/nu15040942 36839299 PMC9965298

[28] Zhang W Wang Y Zhu M Liu K Zhang HL. Gut flora in multiple sclerosis: implications for pathogenesis and treatment. Neural Regener Res. (2024) 19:1480–8. doi: 10.4103/1673-5374.387974 PMC10883522 38051890

[29] Ling Z Cheng Y Yan X Shao L Liu X Zhou D. Alterations of the fecal microbiota in chinese patients with multiple sclerosis. Front Immunol. (2020) 11:590783. doi: 10.3389/fimmu.2020.590783 33391265 PMC7772405

[30] Cantoni C Lin Q Dorsett Y Ghezzi L Liu Z Pan Y. Alterations of host-gut microbiome interactions in multiple sclerosis. EBioMedicine. (2022) 76:103798. doi: 10.1016/j.ebiom.2021.103798 35094961 PMC8814376

[31] Cosorich I Dalla-Costa G Sorini C Ferrarese R Messina MJ Dolpady J. High frequency of intestinal T H 17 cells correlates with microbiota alterations and disease activity in multiple sclerosis. Sci Adv. (2017) 3:e1700492. doi: 10.1126/sciadv.1700492 28706993 PMC5507635

[32] Cekanaviciute E Yoo BB Runia TF Debelius JW Singh S Nelson CA. Gut bacteria from multiple sclerosis patients modulate human T cells and exacerbate symptoms in mouse models. Proc Natl Acad Sci USA. (2017) 114:10713–8. doi: 10.1073/pnas.1711235114 PMC5635915 28893978

[33] Lee YK Menezes JS Umesaki Y Mazmanian SK. Proinflammatory T-cell responses to gut microbiota promote experimental autoimmune encephalomyelitis. Proc Natl Acad Sci USA. (2011) 108 Suppl 1:4615–22. doi: 10.1073/pnas.1000082107 PMC3063590 20660719

[34] Wang S Chen H Wen X Mu J Sun M Song X. The efficacy of fecal microbiota transplantation in experimental autoimmune encephalomyelitis: transcriptome and gut microbiota profiling. J Immunol Res. (2021) 2021:4400428. doi: 10.1155/2021/4400428 34938813 PMC8687821

[35] Lavasani S Dzhambazov B Nouri M Fak F Buske S Molin G. A novel probiotic mixture exerts a therapeutic effect on experimental autoimmune encephalomyelitis mediated by IL-10 producing regulatory T cells. PloS One. (2010) 5:e9009. doi: 10.1371/journal.pone.0009009 20126401 PMC2814855

[36] He B Hoang TK Tian X Taylor CM Blanchard E Luo M. Lactobacillus reuteri reduces the severity of experimental autoimmune encephalomyelitis in mice by modulating gut microbiota. Front Immunol. (2019) 10:385. doi: 10.3389/fimmu.2019.00385 30899262 PMC6416370

[37] Ezendam J de Klerk A Gremmer ER van Loveren H. Effects of Bifidobacterium animalis administered during lactation on allergic and autoimmune responses in rodents. Clin Exp Immunol. (2008) 154:424–31. doi: 10.1111/j.1365-2249.2008.03788.x PMC2633237 19037925

[38] Chen H Ma X Liu Y Ma L Chen Z Lin X. Gut microbiota interventions with clostridium butyricum and norfloxacin modulate immune response in experimental autoimmune encephalomyelitis mice. Front Immunol. (2019) 10:1662. doi: 10.3389/fimmu.2019.01662 31428083 PMC6689973

[39] Bianchimano P Britton GJ Wallach DS Smith EM Cox LM Liu S. Mining the microbiota to identify gut commensals modulating neuroinflammation in a mouse model of multiple sclerosis. Microbiome. (2022) 10:174. doi: 10.1186/s40168-022-01364-2 36253847 PMC9575236

[40] Shahi SK Freedman SN Murra AC Zarei K Sompallae R Gibson-Corley KN. Prevotella histicola, A human gut commensal, is as potent as COPAXONE^®^ in an animal model of multiple sclerosis. Front Immunol. (2019) 10:462. doi: 10.3389/fimmu.2019.00462 30984162 PMC6448018

[41] Shahi SK Jensen SN Murra AC Tang N Guo H Gibson-Corley KN. Human commensal prevotella histicola ameliorates disease as effectively as interferon-beta in the experimental autoimmune encephalomyelitis. Front Immunol. (2020) 11:578648. doi: 10.3389/fimmu.2020.578648 33362764 PMC7759500

[42] Misiak B Loniewski I Marlicz W Frydecka D Szulc A Rudzki L. The HPA axis dysregulation in severe mental illness: Can we shift the blame to gut microbiota? Prog Neuropsychopharmacol Biol Psychiatry. (2020) 102:109951. doi: 10.1016/j.pnpbp.2020.109951 32335265

[43] Wunsch M Jabari S Voussen B Enders M Srinivasan S Cossais F. The enteric nervous system is a potential autoimmune target in multiple sclerosis. Acta neuropathologica. (2017) 134:281–95. doi: 10.1007/s00401-017-1742-6 28620692

[44] Spear ET Holt EA Joyce EJ Haag MM Mawe SM Hennig GW. Altered gastrointestinal motility involving autoantibodies in the experimental autoimmune encephalomyelitis model of multiple sclerosis. Neurogastroenterol Motil. (2018) 30:e13349. doi: 10.1111/nmo.13349 29644797 PMC6153444

[45] Rothhammer V Borucki DM Tjon EC Takenaka MC Chao CC Ardura-Fabregat A. Microglial control of astrocytes in response to microbial metabolites. Nature. (2018) 557:724–8. doi: 10.1038/s41586-018-0119-x PMC6422159 29769726

[46] Thaiss CA Zmora N Levy M Elinav E. The microbiome and innate immunity. Nature. (2016) 535:65–74. doi: 10.1038/nature18847 27383981

[47] Song S Huang H Guan X Fiesler V Bhuiyan M Liu R. Activation of endothelial Wnt/beta-catenin signaling by protective astrocytes repairs BBB damage in ischemic stroke. Prog Neurobiol. (2021) 199:101963. doi: 10.1016/j.pneurobio.2020.101963 33249091 PMC7925353

[48] Campbell A Gdanetz K Schmidt AW Schmidt TM. H2 generated by fermentation in the human gut microbiome influences metabolism and competitive fitness of gut butyrate producers. Microbiome. (2023) 11:133. doi: 10.1186/s40168-023-01565-3 37322527 PMC10268494

[49] Anderson G Maes M. Gut dysbiosis dysregulates central and systemic homeostasis via suboptimal mitochondrial function: assessment, treatment and classification implications. Curr Top Med Chem. (2020) 20:524–39. doi: 10.2174/1568026620666200131094445 32003689

[50] Wang PF Jiang F Zeng QM Yin WF Hu YZ Li Q. Mitochondrial and metabolic dysfunction of peripheral immune cells in multiple sclerosis. J Neuroinflamm. (2024) 21:28. doi: 10.1186/s12974-024-03016-8 PMC10799425 38243312

[51] Witte ME Mahad DJ Lassmann H van Horssen J. Mitochondrial dysfunction contributes to neurodegeneration in multiple sclerosis. Trends Mol Med. (2014) 20:179–87. doi: 10.1016/j.molmed.2013.11.007 24369898

[52] Holman RT Johnson SB Kokmen E. Deficiencies of polyunsaturated fatty acids and replacement by nonessential fatty acids in plasma lipids in multiple sclerosis. Proc Natl Acad Sci USA. (1989) 86:4720–4. doi: 10.1073/pnas.86.12.4720 PMC287343 2734316

[53] van Meeteren ME Teunissen CE Dijkstra CD van Tol EA. Antioxidants and polyunsaturated fatty acids in multiple sclerosis. Eur J Clin Nutr. (2005) 59:1347–61. doi: 10.1038/sj.ejcn.1602255 16118655

[54] Bjørnevik K Chitnis T Ascherio A Munger KL. Polyunsaturated fatty acids and the risk of multiple sclerosis. Multiple sclerosis. (2017) 23:1830–8. doi: 10.1177/1352458517691150 PMC5494026 28156186

[55] Aristotelous P Stefanakis M Pantzaris M Pattichis CS Calder PC Patrikios IS. The effects of specific omega-3 and omega-6 polyunsaturated fatty acids and antioxidant vitamins on gait and functional capacity parameters in patients with relapsing-remitting multiple sclerosis. Nutrients. (2021) 13. doi: 10.3390/nu13103661 PMC8540949 34684661

[56] Bates D Cartlidge NE French JM Jackson MJ Nightingale S Shaw DA. A double-blind controlled trial of long chain n-3 polyunsaturated fatty acids in the treatment of multiple sclerosis. J Neurol Neurosurg Psychiatry. (1989) 52:18–22. doi: 10.1136/jnnp.52.1.18 2540285 PMC1032650

[57] Adkins Y Soulika AM Mackey B Kelley DS. Docosahexaenoic acid (22:6n-3) ameliorated the onset and severity of experimental autoimmune encephalomyelitis in mice. LIPIDS. (2019) 54:13–23. doi: 10.1002/lipd.12130 30762234

[58] Rezapour-Firouzi S Mohammadian M Sadeghzadeh M Mazloomi E. Effects of co-administration of rapamycin and evening primrose/hemp seed oil supplement on immunologic factors and cell membrane fatty acids in experimental autoimmune encephalomyelitis. GENE. (2020) 759:144987. doi: 10.1016/j.gene.2020.144987 32712065

[59] Ríos-Covián D Rúas-Madiedo P Margollés A Gueimonde M de Los RC Salazar N. Intestinal short chain fatty acids and their link with diet and human health. Front Microbiol. (2016) 7. doi: 10.3389/fmicb.2016.00185 PMC4756104 26925050

[60] Cong J Zhou P Zhang R. Intestinal microbiota-derived short chain fatty acids in host health and disease. Nutrients. (2022) 14. doi: 10.3390/nu14091977 PMC9105144 35565943

[61] Barcutean L Maier S Burai-Patrascu M Farczadi L Balasa R. The immunomodulatory potential of short-chain fatty acids in multiple sclerosis. Int J Mol Sci. (2024) 25:3198. doi: 10.3390/ijms25063198 38542172 PMC10970107

[62] Corrêa-Oliveira R Fachi JL Vieira A Sato FT Vinolo MA. Regulation of immune cell function by short-chain fatty acids. Clin Trans Immunol. (2016) 5:e73. doi: 10.1038/cti.2016.17 PMC4855267 27195116

[63] Brestoff JR Artis D. Commensal bacteria at the interface of host metabolism and the immune system. Nat Immunol. (2013) 14:676–84. doi: 10.1038/ni.2640 PMC4013146 23778795

[64] Liu L Li L Min J Wang J Wu H Zeng Y. Butyrate interferes with the differentiation and function of human monocyte-derived dendritic cells. Cell Immunol. (2012) 277:66–73. doi: 10.1016/j.cellimm.2012.05.011 22698927

[65] Morrison DJ Preston T. Formation of short chain fatty acids by the gut microbiota and their impact on human metabolism. Gut Microbes. (2016) 7:189–200. doi: 10.1080/19490976.2015.1134082 26963409 PMC4939913

[66] Zhang LS Davies SS. Microbial metabolism of dietary components to bioactive metabolites: opportunities for new therapeutic interventions. Genome Med. (2016) 8:46. doi: 10.1186/s13073-016-0296-x 27102537 PMC4840492

[67] Stockinger B Shah K Wincent E. AHR in the intestinal microenvironment: safeguarding barrier function. Nat Rev Gastroenterol Hepatol. (2021) 18:559–70. doi: 10.1038/s41575-021-00430-8 PMC7611426 33742166

[68] Rooks M Garrett WS. Gut microbiota, metabolites and host immunity. Nat Rev Immunol. (2016) 16:341–52. doi: 10.1038/nri.2016.42 PMC5541232 27231050

[69] Dunalska A Saramak K Szejko N. The role of gut microbiome in the pathogenesis of multiple sclerosis and related disorders. Cells. (2023) 12:1760. doi: 10.3390/cells12131760 37443793 PMC10341087

[70] Tremlett H Bauer KC Appel-Cresswell S Finlay BB Waubant E. The gut microbiome in human neurological disease: A review. Ann Neurol. (2017) 81:369–82. doi: 10.1002/ana.24901 28220542

[71] Correale J Hohlfeld R Baranzini SE. The role of the gut microbiota in multiple sclerosis. Nature reviews. Neurology. (2022) 18:544–58. doi: 10.1038/s41582-022-00697-8 35931825

[72] Sanchez J DePaula-Silva AB Libbey JE Fujinami RS. Role of diet in regulating the gut microbiota and multiple sclerosis. Clin Immunol. (2022) 235:108379. doi: 10.1016/j.clim.2020.108379 32156562 PMC7483914

[73] Kantor D Sotirchos ES Calabresi PA. Safety and immunologic effects of high- vs low-dose cholecalciferol in multiple sclerosis. Neurology. (2016) 87:1424. doi: 10.1212/01.wnl.0000502811.31151.c9 27672169

[74] Qu ZX Dayal A Jensen MA Arnason BG. All-trans retinoic acid potentiates the ability of interferon beta-1b to augment suppressor cell function in multiple sclerosis. Arch Neurol. (1998) 55:315–21. doi: 10.1001/archneur.55.3.315 9520005

[75] Haghikia A Jörg S Duscha A Berg J Manzel A Waschbisch A. Dietary fatty acids directly impact central nervous system autoimmunity via the small intestine. Immunity. (2016) 44:951–3. doi: 10.1016/j.immuni.2016.04.006 27096322

[76] Aktaş O Prozorovski T Smorodchenko A Savaskan NE Lauster R Kloetzel PM. Green tea epigallocatechin-3-gallate mediates T cellular NF-κB inhibition and exerts neuroprotection in autoimmune encephalomyelitis. J Immunol. (2004) 173:5794–800. doi: 10.4049/jimmunol.173.9.5794 15494532

[77] Berer K Martínez I Walker A Kunkel B Schmitt-Kopplin P Walter J. Dietary non-fermentable fiber prevents autoimmune neurological disease by changing gut metabolic and immune status. Sci Rep. (2018) 8:10431. doi: 10.1038/s41598-018-28839-3 29993025 PMC6041322

[78] James E Dobson R Kuhle J Baker D Giovannoni G Ramagopalan SV. The effect of vitamin D-related interventions on multiple sclerosis relapses: a meta-analysis. Multiple Sclerosis J. (2013) 19:1571–9. doi: 10.1177/1352458513489756 23698130

[79] Runia TF Hop WC de Rijke YB Hintzen RQ. Vitamin A is not associated with exacerbations in multiple sclerosis. Multiple Sclerosis Related Disord. (2014) 3:34–9. doi: 10.1016/j.msard.2013.06.011 25877971

[80] Sato F Martinez NE Shahid M Rose JW Carlson NG Tsunoda I. Resveratrol exacerbates both autoimmune and viral models of multiple sclerosis. Am J Pathol. (2013) 183:1390–6. doi: 10.1016/j.ajpath.2013.07.006 PMC3814682 24091251

[81] Benech N Kapel N Sokol H. Fecal microbiota transplantation for ulcerative colitis. JAMA. (2019) 321:2240. doi: 10.1001/jama.2019.3946 31184729

[82] Belvončíková P Marônek M Gardlík R. Gut dysbiosis and fecal microbiota transplantation in autoimmune diseases. Int J Mol Sci. (2022) 23:10729. doi: 10.3390/ijms231810729 36142642 PMC9503867

[83] Al-Ghezi ZZ Busbee PB Alghetaa H Nagarkatti PS Nagarkatti M. Combination of cannabinoids, delta-9-tetrahydrocannabinol (THC) and cannabidiol (CBD), mitigates experimental autoimmune encephalomyelitis (EAE) by altering the gut microbiome. Brain Behavior Immun. (2019) 82:25–35. doi: 10.1016/j.bbi.2019.07.028 PMC6866665 31356922

[84] Makkawi S Cámara-Lemarroy CR Metz LM. Fecal microbiota transplantation associated with 10 years of stability in a patient with SPMS. Neurology: Neuroimmunol Neuroinflamm. (2018) 5:e459. doi: 10.1212/NXI.0000000000000459 PMC5882466 29619403

[85] Engen PA Zaferiou A Rasmussen H Naqib A Green SJ Fogg LF. Single-arm, non-randomized, time series, single-subject study of fecal microbiota transplantation in multiple sclerosis. Front Neurol. (2020) 11. doi: 10.3389/fneur.2020.00978 PMC7506051 33013647

[86] Ochoa-Repáraz J Mielcarz DW Haque-Begum S Kasper LH. Induction of a regulatory B cell population in experimental allergic encephalomyelitis by alteration of the gut commensal microflora. Gut Microbes. (2010) 1:103–8. doi: 10.4161/gmic.1.2.11515 PMC3023588 21326918

[87] Ochoa-Repáraz J Mielcarz DW Ditrio LE Burroughs AR Foureau DM Haque-Begum S. Role of gut commensal microflora in the development of experimental autoimmune encephalomyelitis. J Immunol. (2009) 183:6041–50. doi: 10.4049/jimmunol.0900747 19841183

[88] Metz LM Li D Traboulsee AL Duquette P Eliasziw M Cerchiaro G. Trial of minocycline in a clinically isolated syndrome of multiple sclerosis. N Engl J Med. (2017) 376:2122–33. doi: 10.1056/NEJMoa1608889 28564557

[89] Salminen S Collado MC Endo A Hill C Lebeer S Quigley E. The International Scientific Association of Probiotics and Prebiotics (ISAPP) consensus statement on the definition and scope of postbiotics. Nat Rev Gastroenterol Hepatol. (2021) 18:649–67. doi: 10.1038/s41575-021-00440-6 PMC8387231 33948025

[90] Klingensmith NJ Coopersmith CM. The gut as the motor of multiple organ dysfunction in critical illness. Crit Care Clinics. (2016) 32:203–12. doi: 10.1016/j.ccc.2015.11.004 PMC4808565 27016162

[91] Wang Y Begum-Haque S Telesford KM Ochoa-Reparaz J Christy M Kasper EJ. A commensal bacterial product elicits and modulates migratory capacity of CD39(+) CD4 T regulatory subsets in the suppression of neuroinflammation. Gut Microbes. (2014) 5:552–61. doi: 10.4161/gmic.29797 25006655

[92] Kwon HK Kim GC Kim Y Hwang W Jash A Sahoo A. Amelioration of experimental autoimmune encephalomyelitis by probiotic mixture is mediated by a shift in T helper cell immune response. Clin Immunol. (2013) 146:217–27. doi: 10.1016/j.clim.2013.01.001 23416238

[93] Rezende RM Oliveira RP Medeiros SR Gomes-Santos AC Alves AC Loli FG. Hsp65-producing Lactococcus lactis prevents experimental autoimmune encephalomyelitis in mice by inducing CD4+LAP+ regulatory T cells. J Autoimmun. (2013) 40:45–57. doi: 10.1016/j.jaut.2012.07.012 22939403 PMC3623677

[94] Mirashrafi S Hejazi TS Sarlak F Moravejolahkami AR Hojjati KM Haratian M. Effect of probiotics supplementation on disease progression, depression, general health, and anthropometric measurements in relapsing-remitting multiple sclerosis patients: A systematic review and meta-analysis of clinical trials. Int J Clin Pract. (2021) 75:e14724. doi: 10.1111/ijcp.14724 34379879

