# Corrigendum: Relationship between gut microbiota and multiple sclerosis: a scientometric visual analysis from 2010 to 2023

**DOI:** 10.3389/fimmu.2024.1489276

**Published:** 2024-10-17

**Authors:** Qingrong Ouyang, Hao Yu, Lei Xu, Ming Yu, Yunwei Zhang

**Affiliations:** ^1^ Department of Neurology, Suining Central Hospital, Suining, China; ^2^ Department of Emergency, Suining Central Hospital, Suining, China

**Keywords:** bibliometrics, gut microbiota, multiple sclerosis, scientometric, VOSviewer, CiteSpace

In the published article, there was an error in the author list, and author Hao Yu co-first author was erroneously displayed as the second author. The corrected author list appears below.

“Qingrong Ouyang^1,†^, Hao Yu ^2,†^, Lei Xu^1,^ Ming Yu^1^, Yunwei Zhang^1,*^”

^†^These authors have contributed equally to this work

The authors apologize for this error and state that this does not change the scientific conclusions of the article in any way. The original article has been updated.

